# Electrochemical Activation of Ni–Fe Oxides for the Oxygen Evolution Reaction in Alkaline Media

**DOI:** 10.1021/acscatal.5c02405

**Published:** 2025-06-18

**Authors:** Emily K. Volk, Melissa E. Kreider, Daniella M. Gibson Colón, Magdalena Müller, Svein Sunde, Shaun M. Alia, Stephanie Kwon

**Affiliations:** † Advanced Energy Systems Graduate Program, 3557 Colorado School of Mines, Golden, Colorado 80401, United States; ‡ 53405 Chemistry and Nanoscience Center, National Renewable Energy Laboratory, Golden, Colorado 80401, United States; § Department of Chemistry, University of Puerto Rico - Rio Piedras, San Juan, PR 00925-2537, United States; ∥ Department of Materials Science and Engineering, 8018 Norwegian University of Science and Technology (NTNU), Trondheim N-7491, Norway; ⊥ Department of Chemical and Biological Engineering, Colorado School of Mines, Golden, Colorado 80401, United States

**Keywords:** oxygen evolution reaction, low temperature electrolysis, NiFe_2_O_4_, NiFe oxide, anion exchange membrane electrolyzer

## Abstract

The oxygen evolution reaction (OER) is essential to many key electrochemical devices, including H_2_O electrolyzers, CO_2_ electrolyzers, and metal–air batteries. NiFe oxides have been historically identified as active for the OER, though they have been less studied in their more commercially relevant bulk oxide forms, such as NiFe_2_O_4_. Past works have demonstrated that the initial starting phase of Ni­(Fe) precatalysts can influence their activation to the Ni­(Fe)­OOH active phase, including the rate and degree of conversion, pointing to the necessity of understanding activation protocols and in situ characteristics of catalyst materials at the device level. In this work, we investigate the characteristics of commercially relevant NiFe bulk oxides (NiFe_2_O_4_ and a physical mixture of NiO and γ-Fe_2_O_3_) during multiple activation procedures. Our results demonstrate that significant performance enhancement is observed for these bulk oxides regardless of the Fe incorporation in the initial form (i.e., atomically or macroscopically integrated), leading to significant performance enhancement (up to 30×) over time on stream. We hypothesize that this activation is due to the formation of NiFeOOH active sites on the surface, supported by in situ cyclic voltammetry and Raman spectroscopy results. We further show that not only the starting material but also the method of activation influences the number of Ni­(Fe)­OOH active sites formed and suggest that these sites can be quantified from the Ni^2+^ to Ni^3+^ redox transition using cyclic voltammetry. Broadly, this work demonstrates the necessity of in situ characterization of catalyst materials for cell-level design and testing.

## Graphical abstract

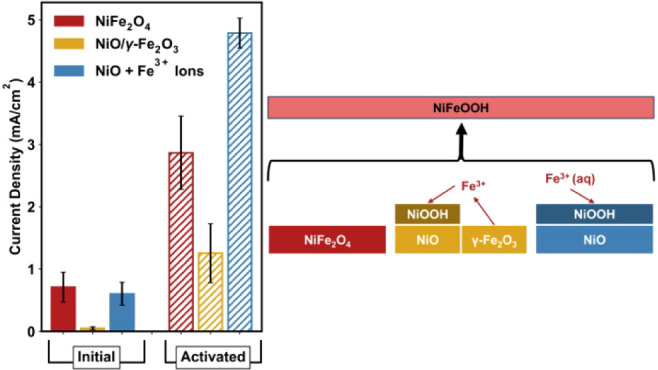

## 1 Introduction

The development of active and stable electrocatalysts for the oxygen evolution reaction (OER) has become essential to facilitate the development and deployment of key electrochemical devices such as water electrolyzers,^1–3^ CO_2_ electrolyzers,^4,5^ and metal–air batteries.^6^ Current state-of-the-art OER catalysts, especially those operating in an acidic near-surface environment, are typically composed of iridium (Ir), platinum (Pt), or ruthenium (Ru) oxides.^1,7^ The use of expensive Pt group metals (PGMs) in electrolyzers is the strongest cost driver outside of economies of scale, but thrifting PGMs to lower the cost can significantly shorten device lifetimes.^1,7^ Consequently, efforts have been made to identify non-PGM catalysts for the OER in alkaline media to lessen these capital costs. NiFe oxides are of particular interest due to their high reported activity for OER^8^ and lower costs and material criticality compared to other PGM and non-PGM alternatives (i.e., IrO_2_, Co_3_O_4_).^9–11^

The important role of Fe in enhancing the activity of Ni catalysts has been widely reported in the literature. These previous studies have shown that even a small incorporation of Fe, either as impurities or intentional additions, can significantly impact the activity of Ni catalysts. Corrigan first reported this phenomenon in 1987 when he observed that Fe impurities in electrolytes significantly boosted the Ni activity for OER.^12^ Later works by Mattinen and Schröder et al.,^13^ Heath et al.,^14^ and Bao et al.^15^ demonstrated that Fe can readily incorporate into the Ni­(OH)_2_/NiOOH structures that form on the surface of Ni metals and oxides at the potentials and pH relevant for OER.^13,15,16^ The activity enhancement by Fe is further supported by the Boettcher group, who showed that Fe incorporated into the crystal structure (i.e., in electrodeposited NiFe­(OH)_2_) can lead to significant performance enhancements.^17,18^ Louie and Bell similarly found that increasing Fe content in electrodeposited NiFe­(OH)_2_/NiFeOOH catalysts led to a linear increase in Ni turnover frequencies, which they hypothesized to be related to Fe altering the redox properties of Ni sites.^19^ This phenomenon has further been found to be unique to Fe; in a study assessing intentionally incorporated metal cations of different identities (i.e., Fe, Mn, La, Ti, Ce), Fe was found to be the only metal that significantly (i.e., several orders of magnitude enhancement) altered the NiOOH catalyst performance.^20^

Few works, however, have investigated the chemistries of commercially available NiFe oxides, notably NiFe_2_O_4_ spinels, that are relevant in industrial applications (i.e., anion exchange membrane water electrolyzers, CO_2_ electrolyzers, or batteries) due to their wide availability and inexpensive synthesis.^21^ These materials have shown high activity for OER in both half-cell and single-cell experiments,^22^ but less is known about their active phases and how they behave in situ. A fundamental understanding of how these catalysts change under operating conditions, including activation/deactivation routes, is critical when considering the likely intermittent operation of electrolyzers coupled to variable renewable energy sources. Previously, we demonstrated that the OER activity of NiFe_2_O_4_ increased during chronoamperometry at 1.8 V, leading to a 70% increase in current density at 1.65 V.^22^ In this work, we expand these findings to investigate the electrochemical activation of NiFe_2_O_4_ spinels over longer time scales and with different activation procedures, as well as to probe the chemical origin of such activation processes.

In this work, the electrochemical activation of NiFe_2_O_4_ was evaluated using a rotating disc electrode (RDE) before and after three different activation procedures: (i) chronoamperometry at 1.8 V (13.5 h), (ii) triangle wave cycling (1.4 to 2.0 V; 30,000 cycles (ν = 750 mV/s); 13.5 h), and (iii) square wave cycling (1.4 to 2.0 V; 1 h/potential; 14 h). The results show that NiFe_2_O_4_ exhibits dramatic activity enhancement by up to 390 ± 50% in 13.5 h (triangle wave cycling) and that the extent of this activation varies based on the activation method. In situ cyclic voltammetry and Raman spectroscopy results indicate the formation of NiFeOOH at high potentials and show that the extent of this phase transformation (and, relatedly, the extent of performance enhancement) depends on the type and duration of the activation procedure. By utilizing the redox peak areas associated with the Ni^2+^/N^i3+^ phase transition (e.g., Ni­(Fe)­(OH)_2_ to Ni­(Fe)­OOH), we show that, for NiFe_2_O_4_, the observed activation is mainly due to the creation of more active sites (i.e., quantity) as opposed to an increase of per-site activity (i.e., quality). We further probed if this activation could occur from similar treatment of a physical mixture of NiO and γ-Fe_2_O_3_, and we show that these physically mixed Ni–Fe catalysts approach similar current densities to NiFe_2_O_4_ upon activation, suggesting the possible incorporation of Fe ions to form NiFeOOH via a dynamic Fe dissolution/redeposition mechanism. Importantly, our findings suggest that not only the nature of parent materials but also the electrochemical testing conditions can drastically influence the quantity and quality of the active phases. These results further show that careful in situ characterization of catalysts is critical for cell-level design and testing.

## 2 Materials and Methods

### 2.1 Catalyst Materials

All catalytic materials were obtained from commercial suppliers and used without further treatment: NiFe_2_O_4_ (US Research Nanomaterials, 98%), NiO (US Research Nanomaterials, 99%), and γ-Fe_2_O_3_ (US Research Nanomaterials Inc., 99%).

### 2.2 Electrode Preparation

Catalyst inks were made with deionized water (Milli-Q; ≥18.2 MΩ resistance and <5 ppb organic carbon content; 76 vol %), *n*-propyl alcohol (24 vol %), and Nafion ionomer (serving as a binding agent). Nafion was added last, after the catalyst/water/nPA solution was allowed to rest in ice for >300 s. Inks were formulated to target 100 μg_metal_/cm^2^ (total metal; i.e., both Ni and Fe) and 0.1 μg_Nafion_/mg_catalyst_. For experiments of physically mixed NiO and γ-Fe_2_O_3_, the molar Ni:Fe ratio was kept consistent with that in the NiFe_2_O_4_ spinel (i.e., Ni:Fe = 1:2). Inks were bath-sonicated for 900 s, horn-sonicated for 60 s, and again bath-sonicated for 1800 s. A total of 20 μL (in two 10 μL depositions) was deposited onto Au RDE working electrodes (polished with Alumina MicroPolish, 0.3 μm) with a 0.1963 cm^2^ active area, rotating at 100 rpm. The rotation rate was increased to 600 rpm for drying (1800 s).

### 2.3 Electrochemical Tests

All catalytic materials were tested in a Teflon electrochemical cell equipped for RDE experiments with a Au mesh counter electrode and a Hg/HgO reference electrode (Mercury Oxide Electrode Kit, 5088 Series, Koslow Scientific). The reference electrode was calibrated prior to every electrochemical test (details in Section S1) and all potentials are reported vs RHE. Overpotentials are reported vs the thermodynamic potential, which was adjusted to account for the elevation in the Denver area (1.224 V), as discussed previously.^23^ All Tafel analysis and activity measurements reported here are *iR*-corrected; ohmic losses from solution resistance through the electrolyte were corrected in situ using values obtained from a built-in current interrupter. We estimated the Tafel slopes as the tangents to the polarization curves at low current densities (between 1.0 and 1.4 mA/cm^2^).

Three different overnight activation procedures were employed in this analysis: 1.8 V hold, 30,000 triangle wave cycles from 1.4 to 2.0 V (ν = 750 mV/s), and square wave potential cycles from 1.4 to 2.0 V (1 h hold at each potential), as shown schematically in Figure S1. Typical testing included 1.0–1.5 h at open circuit potential, followed by 13.5 h (1.8 V hold, triangle wave cycles) or 14 h (square wave cycles) of activation. Extended durability testing for 1.8 V hold and triangle wave cycling was performed for 80–120 h. All electrochemical tests were performed in 0.1 M NaOH (130 mL) electrolyte, which was purged with N_2_ for ≥600 s to remove dissolved gases (i.e., O_2_, CO_2_). After activation and before post test characterization, the electrode was rinsed with deionized water and allowed to dry completely, the electrolyte was refreshed, and the reference electrode was recalibrated. Initial and post-activation performances were characterized by linear sweep voltammetry (LSV; ν = 20 mV/s, RDE rotation rate: 2500 rpm) from 1.2 to 2.0 V and cyclic voltammetry at three scan rates (100, 50, and 20 mV/s) between 0 and 1.6 V. LSVs were collected before CVs and were preceded by five cycles from 1.2 to 1.8 V (100 mV/s, 2500 rpm) to condition the catalyst and check for hysteresis. We expect that any depletion of surface oxygen sustained during CVs would be very small and easily reversed during the activation procedures due to the nature of the time scales involved, similar to how we have shown previously for IrO_2_ OER catalysts;^24^ CVs were collected over ∼15 min from 0.0 to 1.6 V vs 13.5–14 h at oxidative potentials during activation. For experiments with Fe ions, a stock 2 mM solution of Fe­(NO_3_)_3_ hydrate (Thermo Scientific, 99.999% (metals basis), Lot: T11F006) was prepared in water and diluted to a final concentration of 20 μM in a 0.1 M NaOH electrolyte.

### 2.4 In Situ Raman Spectroscopy

In situ Raman spectra were collected at the Norwegian University of Science and Technology (NTNU) using a custom Teflon cell with a quartz window and a three-electrode configuration. All working electrodes were prepared following the same method as described above, and Hg/HgO and Pt mesh were used as the reference and counter electrodes, respectively. A WITec alpha300 R confocal Raman microscope equipped with a 532 nm laser (5 mW) coupled with a Zeiss EC Epiplan 10× objective (G1:600 g/mm, BLZ = 500 nm grating) was used to collect the Raman spectra, as reported previously.^25^ The Raman spectra were processed and analyzed using our in-house Python code. The spectra were smoothed by using a Savitzky–Golay filter with a window length of 50 and a polynomial order of 4. The results were deconvoluted into three Gaussian–Lorentzian peaks at 490, 575, and 690 cm^–1^ with a dynamic baseline drawn between 400 and 800 cm^–1^.

### 2.5 Ex Situ Characterization of Tested Catalysts

X-ray diffractograms (XRD) were obtained (2θ = 13.5–88°) using a Bruker D8 Discover with Cu Kα radiation (γ = 1.5406) and a GADDS XRD system. Inductively coupled plasma-mass spectrometry (ICP-MS) analysis was performed with a Thermo Scientific iCAP Q instrument in kinetic energy discrimination mode using a He cell gas. Samples collected from the electrolyte reservoir were diluted 10 times with 2% HNO_3_ (Fisher Chemical, Optima grade, 67–70%) to obtain a final NaOH concentration of 0.01 M.

## 3 Results and Discussion

### 3.1 Electrochemical Activation of NiFe_2_O_4_

Figure 1a shows the OER activities of NiFe_2_O_4_ in the Tafel plots. Here, the current was normalized by the geometric surface area of the electrode (mA/cm_geo_^2^); we will later discuss how to account for changes in the number of active sites for a more rigorous normalization (in Section 3.2). For a facile activity comparison, the current at 1.65 V (shown as a dashed gray line in Figure 1a) was measured and is shown in Figure 1b; this potential represents the kinetic region for NiFe_2_O_4_, as reported in our previous work.^22^ The measured current density at 1.65 V (J@1.65) was 0.7 ± 0.2 mA/cm^2^ for NiFe_2_O_4_ before any activation procedure (green bar, Figure 1b).

**Figure 1:**
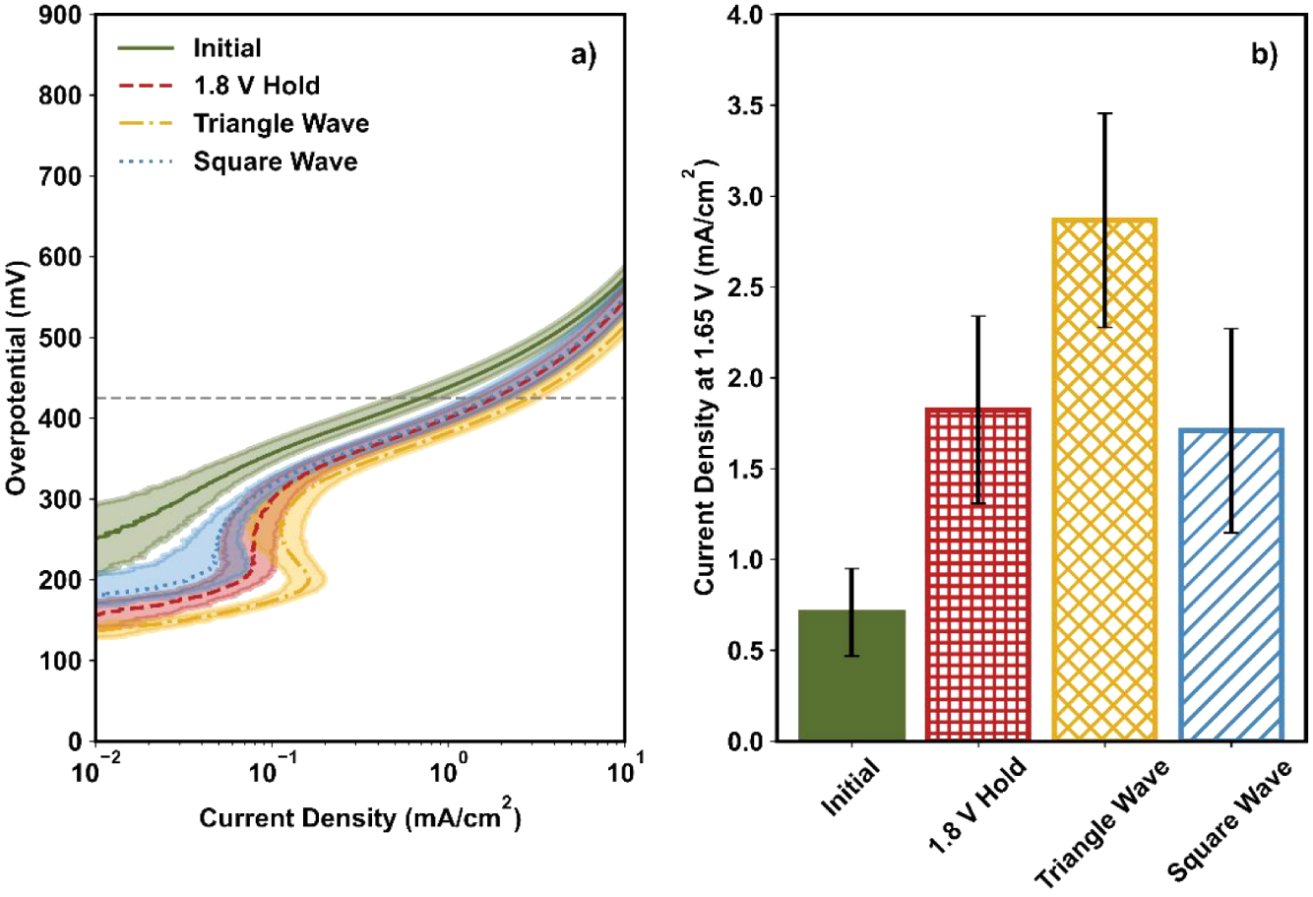
(a) Tafel plot and (b) current density at 1.65 V before and after three different activation procedures for NiFe_2_O_4_. In Figure a, LSVs were collected at a scan rate of 20 mV/s with an RDE rotation rate of 2500 rpm. The dashed gray line is at 1.65 V. The line and the shaded region represent the average and standard deviation, respectively, of three independent experiments. In Figure b, the bar and error bars represent the average and standard deviation, respectively, of three independent experiments.

Next, we tested three different activation protocols to assess their impact on the OER activity of NiFe_2_O_4_. In the first activation protocol, chronoamperometry at 1.8 V (13.5 h) was designed to assess the behavior of the catalyst at potentials relevant to anodes in anion exchange membrane water electrolyzers (AEMWEs).^21^ After the 1.8 V hold, a new curved feature appeared in the Tafel plot at ∼0.1 mA/cm^2^ (red dashed curve, Figure 1a); such a feature has been observed in our previous work and attributed to the Ni^2+^ to Ni^3+^ redox transition,^22^ which will be discussed further alongside the cyclic voltammetry results. After activation, J@1.65 increased from 0.7 ± 0.2 to 1.8 ± 0.5 mA/cm^2^, representing an enhancement of 170 ± 30%. These results led us to hypothesize that the exposure of NiFe_2_O_4_ catalysts to high potential influences their activity, possibly by the formation of additional active sites.

To investigate this further, we assessed the effects of forcing the catalyst through repeated redox transitions via potential cycling. We utilized a triangle wave cycling method where the voltage was swept from 1.4 to 2.0 V via 30,000 (30k) triangle wave cycles for 13.5 h. Such voltage cycling has been used previously as a method to “condition” catalysts through repeated redox transitions, such as in proton exchange membrane (PEM) water electrolysis, where such cycling has been shown to improve cell kinetics, induce polymer creep, and minimize interfacial electronic/ionic resistances.^26^ After triangle wave cycling, lower overpotentials across current density regimes and an additional growth in the curved feature at ∼0.1 mA/cm^2^ (related to the Ni^2+^/Ni^3+^ redox transition) were observed relative to the results for the 1.8 V hold (red dashed vs yellow dash-dot curves; Figure 1a). Furthermore, the J@1.65 increased to 2.9 ± 0.6 mA/cm^2^ after the triangle wave cycles (yellow bar, Figure 1b), reflecting a larger enhancement of 390 ± 50% compared to that of the 1.8 V hold (170 ± 30%).

To complement these results, square wave potential cycling was selected to assess any difference between dynamic, rapid triangle wave cycling, and longer chronoamperometric holds (1 h) at high (2.0 V) and low (1.4 V) potentials. Interestingly, after square wave cycling, the behavior after activation was very similar to that of the 1.8 V hold procedure; these materials had similar overpotentials across current density regimes (red dashed line and dotted blue line, Figure 1a) and similar values for J@1.65 of 1.8 ± 0.5 mA/cm^2^ (1.8 V hold) and 1.7 ± 0.6 mA/cm^2^ (square wave cycles; red vs blue bars; Figure 1b) despite differences in the potentials applied.

Broadly, these results demonstrate that the activation of NiFe_2_O_4_ is universal across activation procedures. Yet, we found that the type of activation procedure affected the degree to which this activation occurs, which may reflect the differences in (i) the number of active sites (i.e., active site quantity) and/or (ii) the chemical composition/structure of the active phase (i.e., active site quality), depending on the activation procedure. We next aim to identify the origins of NiFe_2_O_4_ activation and answer the question of why different activation procedures lead to different extents of activation.

First, we explored the possibility that NiFe_2_O_4_ activation was driven by a change in the chemical composition, possibly caused by Fe dissolution. Fe inclusion in Ni-based materials has been suggested to facilitate preferred M–O_*x*_H_*y*_ binding energies,^27^ improve the electronic conductivity of NiFe oxide catalysts,^17^ or change the electronic properties of Ni sites, delaying the Ni^2+^/Ni^3+^ transition and lowering the overall oxidation state.^19^ Fe, however, does not itself have high OER activity; our previous work has shown that γ-Fe_2_O_3_ exhibits poor activity toward OER, with overpotentials over 100 mV higher than NiO or NiFe_2_O_4_.^22^ There is therefore a reported “optimal” Fe content for NiFe oxide catalysts related to the optimization of the above characteristics, which is often suggested to be between 5 and 40 wt % Fe.^17,19,28^ The NiFe_2_O_4_ spinels used in this work, however, contain 66 wt % Fe; therefore, Fe dissolution could improve activity by producing a more “optimal” Ni:Fe ratio.

To test our hypothesis, we performed posttest ICP-MS measurements to quantify Fe dissolution after each activation procedure. For the 1.8 V hold test, no Fe dissolution was detected; Fe levels in the electrolytes were within the background equivalent concentration (BEC ∼2 ppb). For both the triangle wave cycles and the square wave cycles, similar dissolution was observed (∼5% Fe loss) (Figure S2). While cycling appears to induce more Fe dissolution than voltage holds, these relatively low levels of dissolution represent a small composition change and are unlikely to account for the significant activity increase observed in Figure 1. Additionally, local compositional changes driven by Fe loss at the surface are not expected, as there were negligible changes to the Fe redox features observed in cyclic voltammetry (discussed in more detail below). Furthermore, ex situ X-ray diffractograms of samples before and after 1.8 V-hold and 1.4–2.0 V cycling tests yielded no observable change in the bulk NiFe_2_O_4_ phase (Figure S2). These results collectively suggest that the observed activity enhancement is not directly related to changes in the Fe content or bulk structure of the material.

We next hypothesized that there may be a change in surface chemistry driving the observed performance trends, which can be related to the quality of the active sites (i.e., a generation of new phases upon activation) and/or the quantity of active sites (i.e., the nature of active sites remains the same, but the activation procedure generates more of them).

Figure 2 shows cyclic voltammograms of NiFe_2_O_4_ before and after each activation procedure. In these voltammograms, several notable features are observed; these features are discussed here in order of increasing potential. An oxidative peak assigned to the Fe^2+^ to Fe^3+^ transition was observed between 0.0 and 0.2 V for all three tests, which remained generally unchanged after the activation procedures.^29^ This finding suggests that no additional Fe sites were formed or exposed during testing and that subsurface Fe oxide growth was not so extensive enough to add to the electrochemical hysteresis or change the Coulombic charge associated with the Fe^2+^ to Fe^3+^ transition.

**Figure 2:**
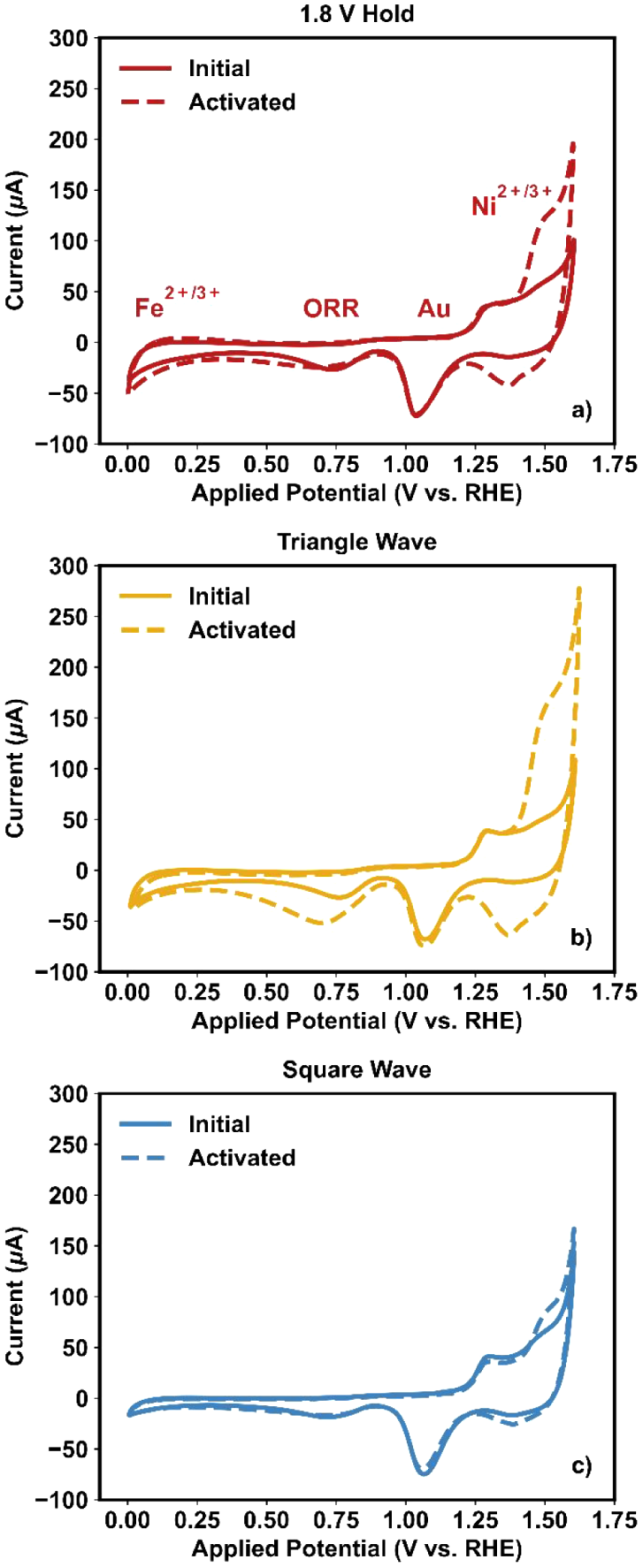
Cyclic voltammograms of NiFe_2_O_4_ before and after (a) 1.8 V hold, (b) triangle wave cycles, and (c) square wave cycles. Scan rate: 100 mV/s.

At 0.8 V, a reduction feature was observed, which most likely originates from the current toward oxide reduction and/or the oxygen reduction reaction on the Au electrode substrate;^30^ O_2_ generated during activation or activity screening (LSVs) could have been left on the surface, fueling this reaction. The most active catalysts (i.e., after triangle wave cycling) showed the highest peak within this regime (i.e., the yellow dashed curve in Figure 2b). The pair of peaks between 1.0 and 1.2 V are assigned to the oxidation (and subsequent reduction) of the Au substrate.^31^

Lastly, between 1.4 and 1.5 V, a pair of peaks is observed in all samples assigned to the redox transition between Ni^2+^ and Ni^3+^.^13,19,32^ In all cases, there was an observed increase in the peak height for this feature after the activation procedure, indicating an increase in the number of Ni sites participating in the Ni^2+^ to Ni^3+^ redox transition. Differences in the resolution of the Fe^2+^/Fe^3+^ and Ni^2+^/Ni^3+^ transitions may be due to the differences in the Coulombic charge transfer and the fact that the Fe redox transition tends to distribute across a larger potential range, as shown previously.^19^

This Ni^2+^/Ni^3+^ redox transition is of particular importance to Ni and NiFe oxide electrochemistry. Past works have assigned this peak to the transition from Ni^2+^(OH)_2_ to Ni^3+^OOH.^13,17,19^ Past works have found an average of ∼1.3–1.7 mol e^–^/Ni transfer during this transition,^13–35^ and some authors have suggested that this indicates the further oxidation of Ni and the formation of ∼3.5+ or 4+ species of Ni.^35^ Generally, this redox feature is considered to be an indicator of the transition from Ni^2+^(OH)_2_ to Ni^3+^OOH. Such a phase transition does not involve a change in the Fe redox state, consistent with the lack of change in the size of the Fe feature discussed above. As this Ni^3+^OOH phase is reported to be the more active form for OER,^13,15,17,19^ this Ni^2+^/Ni^3+^ transition process has been often denoted as “the electrochemical aging of Ni”.^16^ Mattinen and Schröder et al. recently demonstrated that the degree to which Ni-based precatalyst materials convert to NiOOH depends on the initial starting phase and composition, as well as the degree of Fe inclusion.^13^ Two important conclusions can be inferred from these literature insights: first, the formation of Ni^3+^ is important for the OER catalysis and this is associated with NiOOH formation on Ni metal and oxide catalysts. For NiFe_2_O_4_, we hypothesize that this redox feature could be comparably related to NiFeOOH formation, as corroborated by our Raman spectroscopy data, discussed next. Second, Ni­(Fe)­OOH sites can be quantified by measuring the number of Ni atoms involved in the Ni^2+^/Ni^3+^ redox transition; this was leveraged in our assessment of the active sites discussed in Section 3.2.

To validate the formation of the NiFeOOH active phase on the NiFe_2_O_4_ surface, in situ Raman spectroscopy was employed; the results are shown in Figure 3. A detailed schematic illustrating the experimental method can be found in Figure S3. The black curve represents the ex situ Raman spectra of the NiFe_2_O_4_ powder, with peaks at 295, 460, 540, and 675 cm^–1^. These results are consistent with those reported in the literature and correspond to the Eg (Ni–O or Fe–O), T2g (2) and T2g (3) (Ni–O or Fe–O in octahedral sites), and A1g (Fe–O in tetrahedral sites) modes, respectively.^36^ Upon submersion in the electrolyte and at open circuit potential (OCP; gray curve), the peaks at 460 and 675 cm^–1^ shifted by +30 and +15 cm^–1^, respectively. Based on prior literature, these new bands at 490 and 690 cm^–1^ are assigned to Ni–O^19,37^ and Fe–O^36^ in Ni­(Fe)­(OH)_2_, respectively, suggesting the adsorption of OH^–^ species from the electrolyte and the formation of Ni­(Fe)­(OH)_2_.^19^ Past works have shown that the oxidation of Ni to Ni­(OH)_2_ is relatively facile and can proceed readily without an applied potential, supporting this claim.^15,19,38^

**Figure 3:**
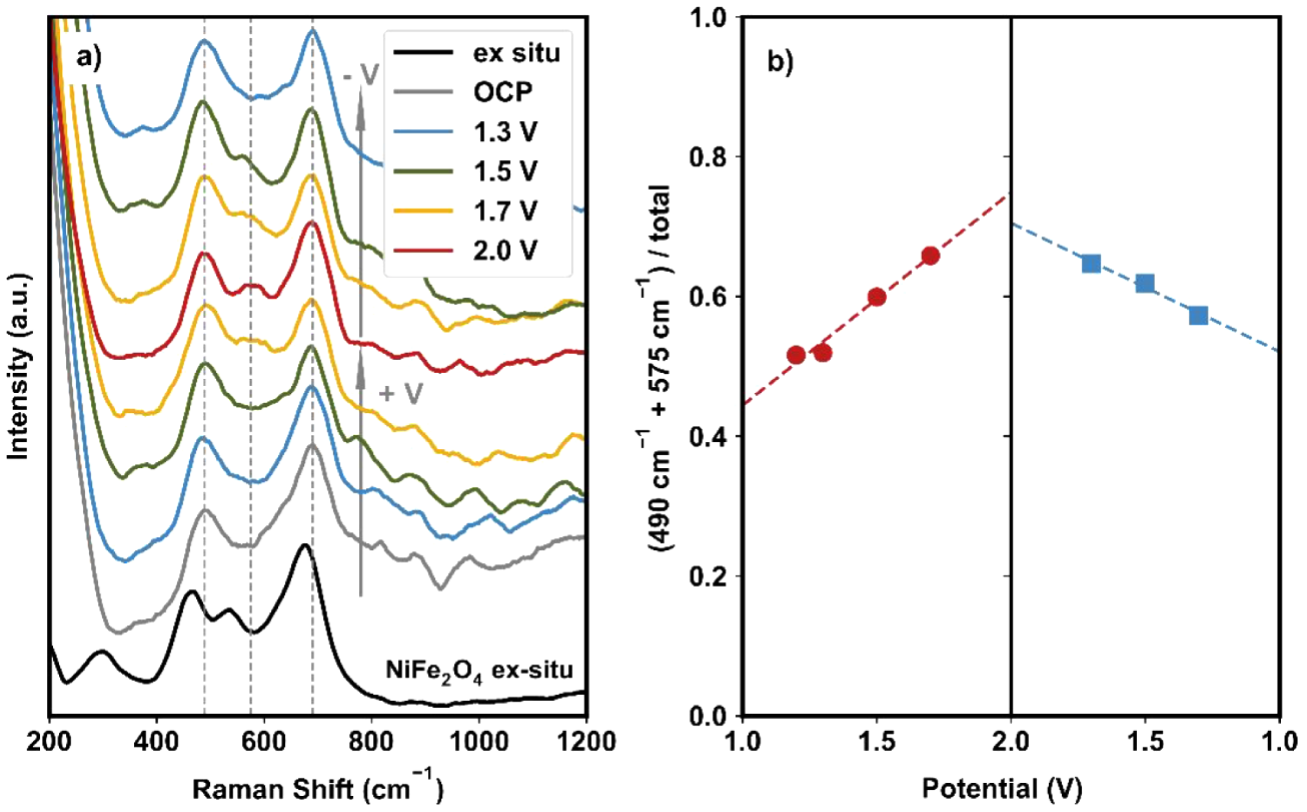
(a) Raman spectra for NiFe_2_O_4_. From bottom to top: the black curve shows the ex situ Raman spectra of the untested powder; the gray curve represents the spectrum collected at the OCP; and the blue, green, yellow, and red curves show the spectra at increasing applied voltages (1.3–2.0 V). The following yellow, green, and blue curves are the spectra collected at decreasing voltages. (b) The ratio of the areas of the peaks at 490 + 575 cm^–1^ to the total (490 + 575 + 690 cm^–1^) for the anodic (increasing) and cathodic (decreasing) voltage sweeps.

As the potential increased incrementally, a third peak at 575 cm^–1^ became more apparent (as can be seen clearly for the 2.0 V spectrum; red curve, Figure 3a), concurrent with an increase in the relative intensity of the peak at 490 cm^–1^. This pair of peaks (at 490 and 575 cm^–1^) has been previously attributed to Ni–O bands in Ni­(Fe)­OOH,^19,37–39^ and this result suggests the formation of Ni­(Fe)­OOH on NiFe_2_O_4_ at conditions relevant to OER catalysis. Figure 3b shows the relative intensities of this pair of peaks at 490 cm^–1^ and 575 cm^–1^ compared to the total area of the three peaks between 400 and 800 cm^–1^ (490, 575, and 690 cm^–1^). As the potential was increased, the relative intensity of the peaks at 490 cm^–1^ and 575 cm^–1^ increased linearly with increasing applied potential. As the potential was decreased back toward the OCP, the relative intensity of these peaks also decreased linearly, suggesting that this phase transformation is quasi-reversible and can only be observed in situ, emphasizing the need for in situ characterization to understand the active phases on NiFe oxides. The surface phase was not completely restored, however, as we discuss further in the context of the extended durability tests presented in Section 3.2. From past studies evaluating metal/oxide transitions, we believe that this hysteresis likely arises from the surface oxidizing at a faster rate than it can be reduced.^24^ While our understanding of this hysteresis is qualitative, it does suggest that the formation of NiFeOOH is not fully reversible within the duration of these experiments. This, and evaluations of the ratio of Ni–O to Fe–O features with different catalyst compositions and activation protocols, could be a valuable avenue for future work. Having established that the activation protocol influences the formation of Ni­(Fe)­OOH, we look to quantify the changes in the number of active sites in the next section and how they relate to the observed activity enhancement.

### 3.2 A Quantification of Changes in the Number of Active Sites during NiFe_2_O_4_ Activation

Figure 4 summarizes the enhancement in the OER activity of NiFe_2_O_4_ following various activation procedures, as indicated by the percentage increase in current density at 1.65 V (J@1.65) before and after activation. The leftmost bars represent J@1.65 values normalized by the geometric surface area of the electrodes, as is common. However, this form of normalization does not capture changes in active surface area or the number of catalytically active sites induced by activation. As such, these values alone cannot distinguish whether improvements arise solely from an increased quantity of active sites (e.g., through greater surface exposure or the formation of more active sites) or from an enhancement in the intrinsic activity (i.e., quality) of each site. If the activation procedures only increase the number of active sites, then the percentage increase in activity per site would approach zero after accurate normalization by active site count. Conversely, if the activity per site remains significantly higher even after such normalization, it would indicate that the activation procedures also improve the intrinsic catalytic performance of the sites themselves. To explore this distinction, we next applied two normalization strategies to account for changes in active surface area and active site density resulting from the activation procedures.

**Figure 4:**
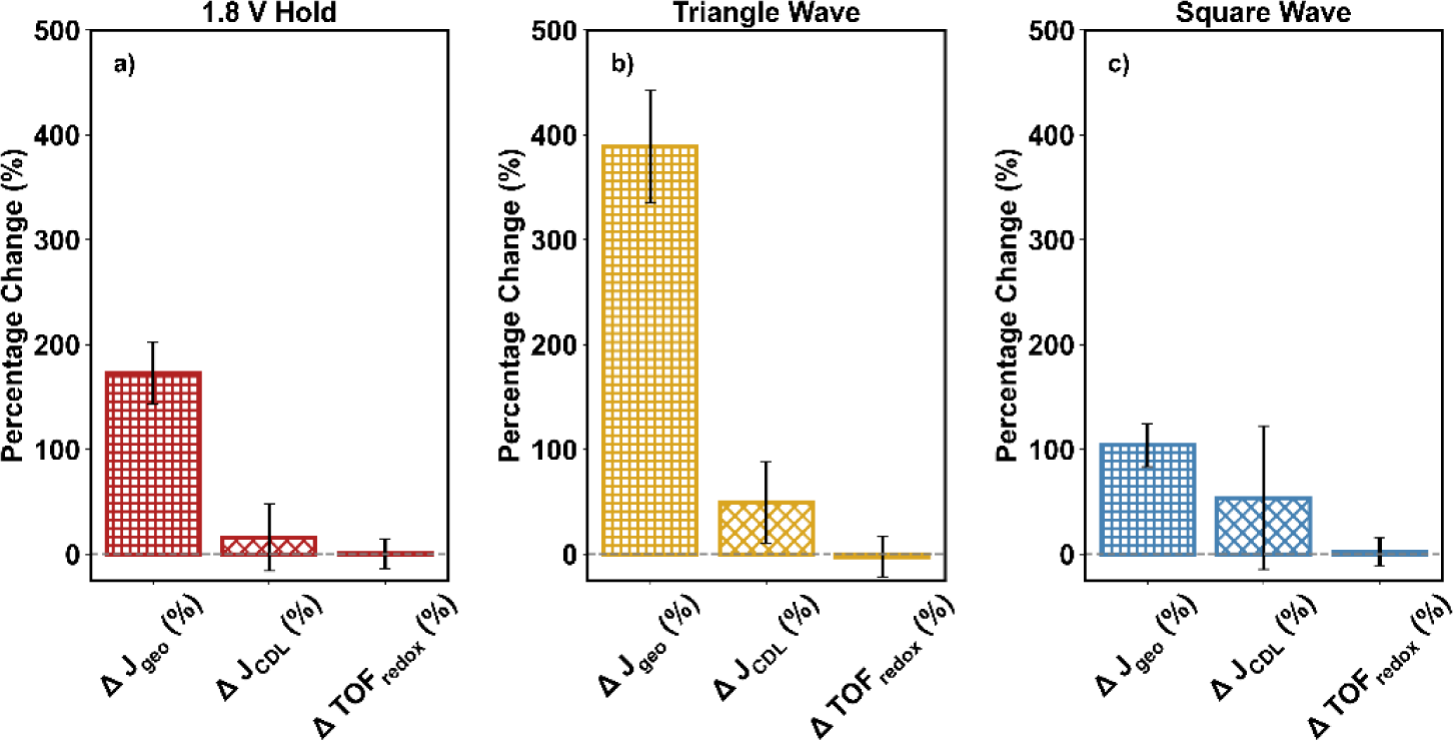
Percentage change in the current density normalized by the geometric surface area of the electrode (Δ*J*_geo_), in the current density normalized by the *C*_DL_ (Δ*J*_CDL_), and in the redox turnover frequency (ΔTOF_redox_) for NiFe_2_O_4_ after (a) 1.8 V hold, (b) triangle wave cycles, and (c) square wave cycles. The bars and error bars represent the average and standard deviation, respectively, of three independent experiments.

The double-layer capacitance (*C*_DL_) is often used as a proxy for the electrochemically active surface area of transition metal oxides; a lack of species that bind selectively and strongly to these oxide surfaces precludes the use of methods involving quantifying adsorbates, such as the CO stripping method used for PGM materials. The *C*_DL_ has been considered to be fairly accurate in quantifying the surface area of materials with the same composition,^40^ though it is only considered to be accurate within an order of magnitude for different materials due to a lack of knowledge on how to accurately determine intrinsic capacitances.^41^ The *C*_DL_ was calculated from cyclic voltammetry at three scan rates (100, 50, and 20 mV/s) as described previously.^22^ For all activation procedures, the *C*_DL_ increased after testing, consistent with an increase in electrochemically active surface area (Figure S4). In Figure 4, normalization by the *C*_DL_ indeed led to a decrease in the percentage increase in current, indicating that the increase in *C*_DL_ is related to the increase in the level of OER activity.

In Section 3.1, we demonstrated from Raman spectra that NiFe_2_O_4_ evolves into Ni­(Fe)­(OH)_2_ and subsequently Ni­(Fe)­OOH, where the latter Ni^2+^/Ni^3+^ transformation occurs only at high potentials. We thus hypothesize that the activation procedures increase the number of Ni sites that participate in the Ni^2+^/Ni^3+^ transition. If the observed activity enhancements are solely caused by the increase in the number of active Ni­(Fe)­OOH sites, then we should therefore be able to rigorously account for these enhancements by accurately quantifying these redox-active Ni sites.

To address this, we quantified the increase in the number of Ni­(Fe)­OOH sites from the number of Ni atoms participating in the Ni^2+^ to Ni^3+^ redox transition, estimated by integrating the reduction peak associated with this transition and converting the peak area to the number of Ni atoms. This then can be used to calculate a turnover frequency (TOF) toward the OER, where

1 $${\mathrm{TOF}}_{\mathrm{redox}}=\frac{\mathrm{molecules}\mathrm{of}O_{2}\mathrm{produced}/s}{\mathrm{molecules}\mathrm{of}\mathrm{redox}\mathrm{active}\mathrm{Ni}}$$

Details of these calculations are in Section S6. This method is similar to that described by Mattinen and Schröder et al. in their study of Ni OER precatalysts.^13^

In Figure 4, the rightmost bar in each subfigure represents the percentage change in the TOF_redox_ for all activation procedures. Here, changes to the amount of redox-active Ni completely account for the increase in activity for all activation procedures. For current normalized by the geometric surface area, the percentage increase in current density was 170 ± 30%, 390 ± 50%, and 100 ± 20% for the 1.8 V, triangle wave cycle, and square wave cycle activation procedures, respectively. When normalized by the change in redox-active Ni sites, however, the percentage increase in current density approached zero at 1 ± 14%, −2 ± 19%, and 2 ± 13% for the 1.8 V, triangle wave cycle, and square wave cycle activation procedures, respectively. This result suggests that for NiFe_2_O_4_, the increase in current density is caused by the creation of additional active sites as opposed to an increase in per-site activity.

This conclusion is further supported by evaluating changes in the kinetic parameters for this material (Figure 5); there was no change in the Tafel slope after activation, indicating that there was no change in the kinetic mechanism or surface coverage during activation (Figure 5a). There was also no change in the exchange current density between the initial state and after activation (Figure 5b), indicating that there was no change in the inherent catalytic activity of the tested samples (i.e., no change in the nature of the active site). These trends are consistent for the exchange current densities normalized by the quantity of redox-active Ni sites (Figure S5), reaffirming this conclusion.

**Figure 5:**
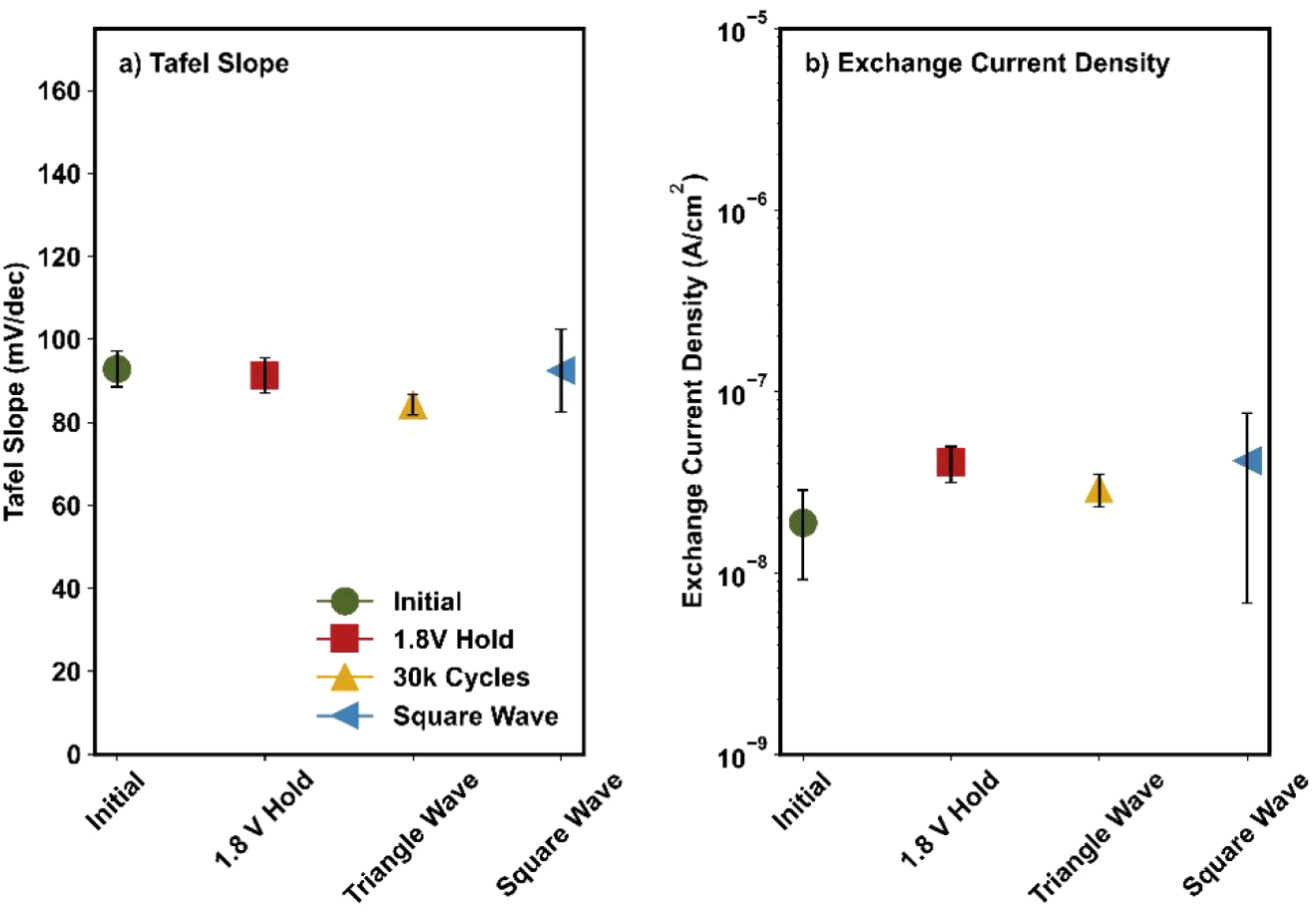
Values for the (a) Tafel slope and (b) exchange current density for NiFe_2_O_4_ before and after the three activation procedures. The points and error bars represent the average and standard deviation, respectively, of three independent experiments.

Next, we conducted extended activation treatments—either by holding at 1.8 V or cycling between 1.4 and 2.0 V (triangle wave)—for up to 120 h to investigate the extent of the reactivity enhancement and the active site formation (Figure 6). Raw cyclic voltammetry and linear sweep voltammetry data are provided in Figure S6. Both the OER activity (J@1.65) and the number of redox-active Ni sites (quantified by the Ni^2+^/Ni^3+^ transition from cyclic voltammetry measurements) increased during the early stage of activation but plateaued after ∼10 h for the 1.8 V hold and ∼20 h for the triangle wave cycling (Figure 6a,b). These data were used to correlate the OER activity with the moles of redox-active Ni in Figure 6c. The observed linear relationship suggests that the enhanced OER activity resulting from the activation procedures is primarily attributable to an increase in the number of active sites, specifically the formation of Ni­(Fe)­OOH.

**Figure 6:**
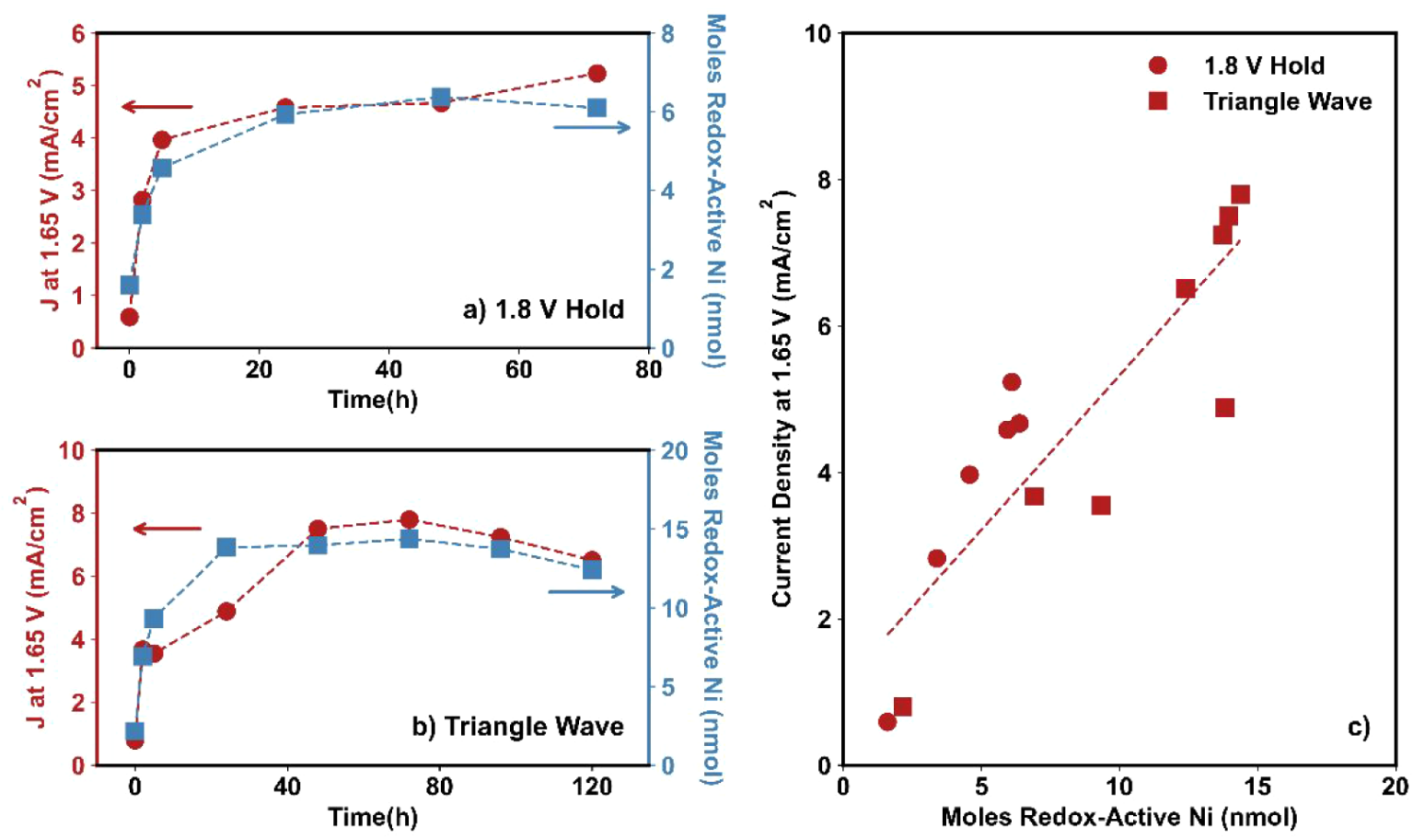
Extended activation results for NiFe_2_O_4_ exposed to a 1.8 V hold and triangle wave cycles (ν = 500 mV/s). (a, b) Current density at 1.65 V (red circles, left axis) and the moles of Ni participating in Ni^2+^/Ni^3+^ transition (blue squares, right axis) shown as a function of activation time for (a) the 1.8 V hold and (b) the triangle wave cycles tests. (c) Current density normalized by the geometric surface area of the electrode vs the number of moles of redox-active Ni for the 1.8 V hold (circles) and 1.4 to 2.0 V cycles (squares) tests. Dashed lines in (a) and (b) are guides to the eye, and the dashed line in (c) represents the best-fit line for the data.

The extent of activation and the number of redox-active Ni sites formed varied significantly between the two activation methods. During the 1.8 V hold test, the current density plateaued at ∼5 mA/cm^2^ and the amount of redox-active Ni reached ∼6 nmol (Figure 6a). In contrast, the triangle wave cycling method resulted in a higher plateau current density of ∼8 mA/cm^2^ and ∼15 nmol of redox-active Ni (Figure 6b). These findings indicate that the activation method influences the extent of phase transformation to the active Ni­(Fe)­OOH phase, likely due to differences in the depth of transformation into the bulk material. When comparing the redox-active Ni to the total Ni content, only ∼7% of Ni participated in the Ni^2+^/Ni^3+^ redox transition during the hold test, whereas ∼17% participated during the cycling test. These values are rough estimates based on redox peak integration and do not fully account for differences in Ni accessibility or oxide composition. Nevertheless, the results qualitatively suggest that the phase transformation is largely confined to the near-surface region and that the extent of penetration—and thus the number of active sites generated—is strongly dependent on the activation method used.

### 3.3 The Role of Ni–Fe Proximity in Activation

In NiFe_2_O_4_, Ni and Fe are in close proximity at the atomic scale, where electronic effects from Fe on Ni (or vice versa) may contribute to activity trends; this bimetallic nature has been suggested to be necessary for high OER activity.^27,42^ Literature insights, however, have also suggested that Ni metal and oxide catalysts can improve in performance dramatically when Fe ions are present in the electrolyte,^12,17^ suggesting that Fe in the solution may be electrochemically depositing under operating conditions. To probe this, we next investigated the electrochemical activation of a physical mixture of NiO and γ-Fe_2_O_3_ (NiO + γ-Fe_2_O_3_) and compared the results to those of NiFe_2_O_4_.

The OER activity for NiO + γ-Fe_2_O_3_ is shown in Figure 7. At the “initial” condition, no distinct features were observed in the Tafel plot (green solid curve, Figure 7a) and the J@1.65 for NiO + γ-Fe_2_O_3_ was much lower than that of the NiFe_2_O_4_ sample (0.05 ± 0.02 mA/cm^2^ for NiO + γ-Fe_2_O_3_ vs 0.7 ± 0.2 mA/cm^2^ for NiFe_2_O_4_; Figures 7b and 1b). This result is consistent with the relatively poor performance of γ-Fe_2_O_3_ individually^22^ and suggests that the Ni and Fe proximity is necessary for high activity.

**Figure 7:**
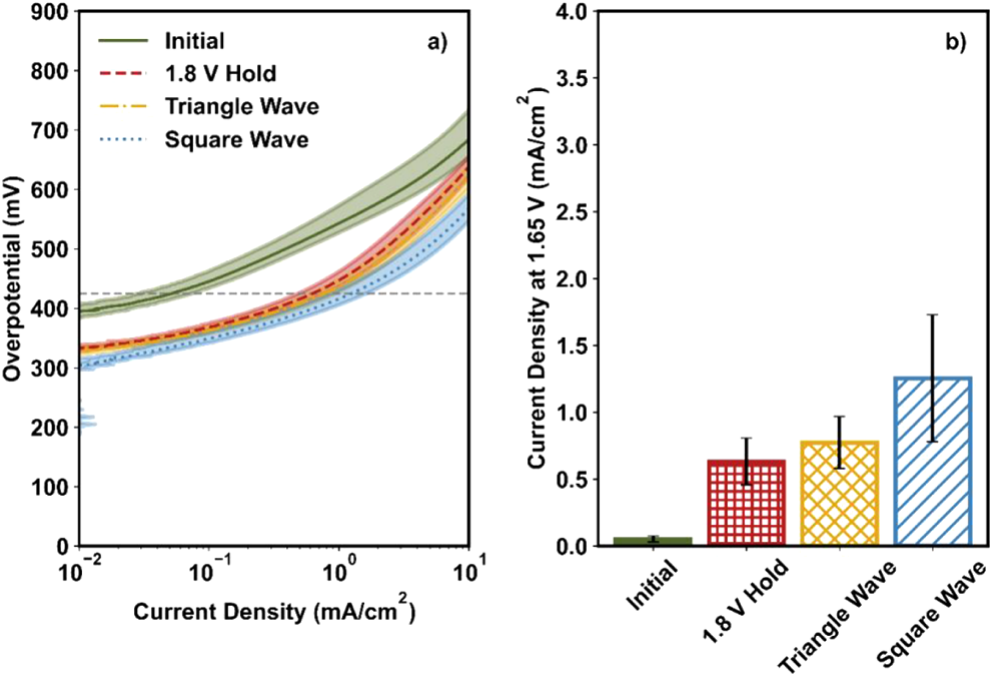
(a) Tafel plot and (b) current density at 1.65 V before and after three different activation procedures for a physical mixture of NiO and γ-Fe_2_O_3_. In Figure a, LSVs were collected at a scan rate of 20 mV/s with an RDE rotation rate of 2500 rpm. The dashed gray line is at 1.65 V. The line and the shaded region represent the average and standard deviation, respectively, of three independent experiments. In Figure b, the bar and error bars represent the average and standard deviation, respectively, of three independent experiments.

After the activation procedure, overpotentials dropped dramatically (by ≥100 mV) across current density regimes (red, yellow, and blue curves in Figure 7a). Furthermore, for all activation procedures, the J@1.65 increased by at least an order of magnitude (Figure 7b), reaching 0.6 ± 0.2 mA/cm^2^ (+1440 ± 440%), 0.8 ± 0.2 mA/cm^2^ (+970 ± 310%), and 1.3 ± 0.5 mA/cm^2^ (+3620 ± 1480%) for the 1.8 V hold, triangle wave cycles, and square wave cycles, respectively. These OER activities were comparable to or larger than those of the NiFe_2_O_4_ catalyst before activation (0.7 ± 0.2 mA/cm^2^) and approached those of activated NiFe_2_O_4_ (1.8 ± 0.5 mA/cm^2^ after the 1.8 V hold; Figure 1b).

As with NiFe_2_O_4_ (discussed in Section 3.2), we quantified the number of redox-active Ni sites in the NiO + γ-Fe_2_O_3_ catalyst to evaluate whether the observed increase in the level of OER activity arose primarily from an increase in the number of active sites or also involved improvements in site quality. When the activity enhancement was normalized by the number of redox-active Ni (ΔTOF_redox_), the percentage increase was smaller than that observed using geometric surface area normalization, but it still did not fully account for the overall performance gain (Figure 8). The percentage increases in ΔTOF_redox_ were 350 ± 120% for the 1.8 V hold, 270 ± 60% for triangle wave cycling, and 940 ± 430% for square wave cycling. These results suggest that the activation procedures not only increased the number of active sites but also may have improved their intrinsic activity. Additional details, including cyclic voltammograms of NiO + γ-Fe_2_O_3_ following each activation method, are provided in Section S8.

**Figure 8:**
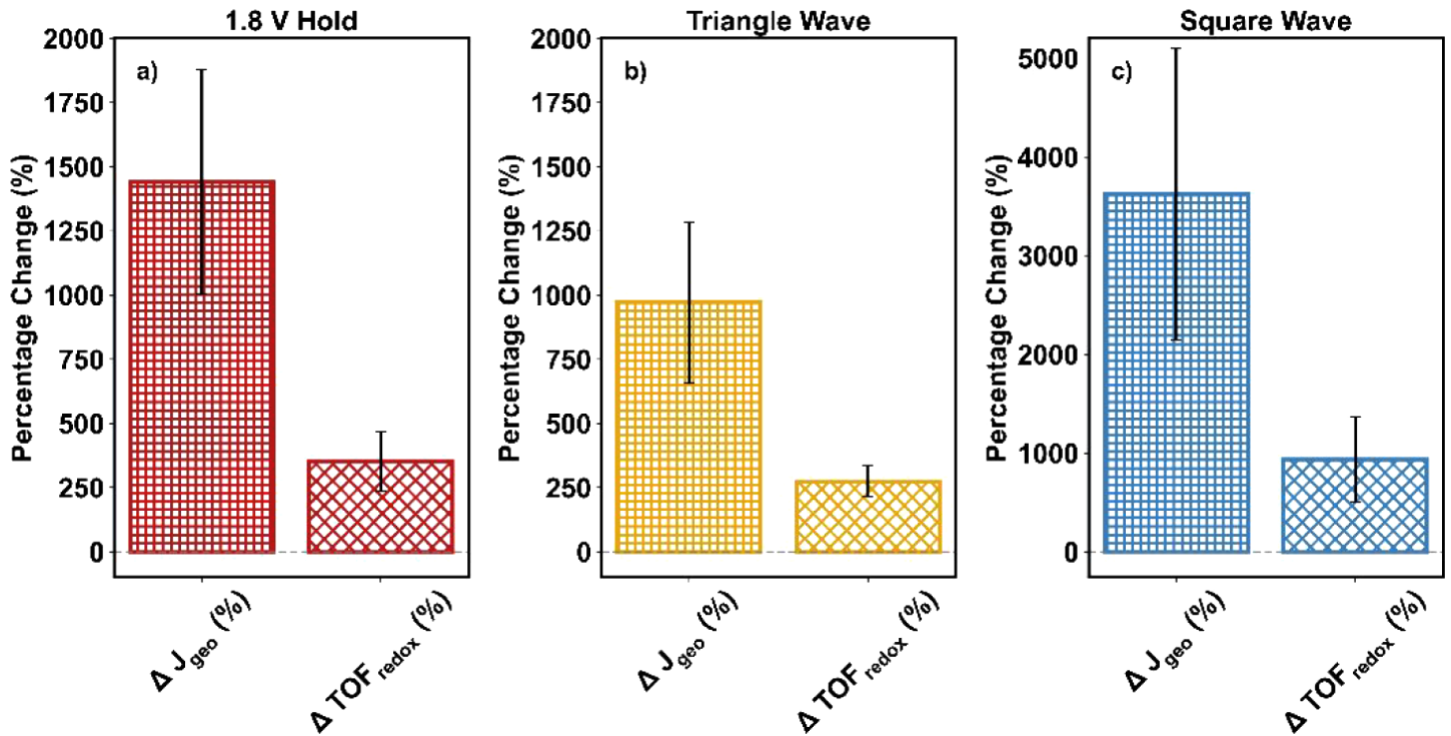
Percentage change in the current density normalized by the geometric surface area of the electrode (Δ*J*_geo_) and in the redox turnover frequency (ΔTOF_redox_) for a physical mixture of NiO and γ-Fe_2_O_3_ after (a) 1.8 V hold, (b) triangle wave cycles, and (c) square wave activation procedures.

These results suggest that there was an increase in active site *quantity* as the percentage change decreased upon normalization by the number of redox-active Ni sites. This can likely be attributed to the conversion of NiO sites to Ni­(OH)_2_ and NiOOH, as has been reported previously.^13,16^ However, this creation of additional redox-active Ni sites did not fully account for the dramatic enhancement of the NiO + γ-Fe_2_O_3_ catalyst, contrary to the result for activated NiFe_2_O_4_. This result suggests that for NiO + γ-Fe_2_O_3_ the increase in activity was due to both an increase in the number of active sites (i.e., quantity) and an increase in the per-site activity (i.e., quality). The trends in the kinetic parameters further support this claim, as seen in Figure S8, where an increase in exchange current density after activation was observed (Figure S8b), suggesting an increase in the per-site activity.

We hypothesized that there was therefore a formation of a new type of active site, Ni­(Fe)­OOH, facilitated by the dissolution of Fe from γ-Fe_2_O_3_ and the incorporation of those Fe^3+^ ions into the Ni­(OH)_2_/NiOOH framework via a mechanism similar to that proposed by Bao et al.^15^ Here, the quantity of active sites increased via the creation of Ni­(OH)_2_/NiOOH from NiO and likely also from the exfoliation of the surface from Fe dissolution (consistent with an increase in *C*_DL_, Figure S4) and the quality of active sites increased via the transformation of Ni­(OH)_2_/NiOOH to NiFe­(OH)_2_/NiFeOOH via Fe redeposition. We note that ICP-MS results after activation for the physically mixed catalyst (Figure S2) indicate negligible Fe dissolution within the background equivalent concentration of our system, suggesting that such a dynamic Fe dissolution/redeposition mechanism likely takes place at the near-surface. In the next section, we explore further this proposed dynamic Fe dissolution/redeposition mechanism via testing of NiO with intentionally added Fe^3+^ in the electrolyte.

### 3.4 The Role of Dissolved Fe on Activation

Fe, in its metallic or oxide forms, is known to dissolve at OER-active conditions.^31^ The dissolution of Fe can increase surface roughness and create surface defects, increasing electrochemically active surface areas and introducing more active sites; Fe edge/defect sites have been suggested to be highly active for OER.^15,43–46^ In parallel, dissolved Fe can redeposit on the electrode surface from the electrolyte and continue to participate in OER.^14,15,43^

Recent works have suggested that a dynamic equilibrium may be established via a dissolution/redeposition mechanism.^15,43,47^ Chung et al. provided direct evidence of this mechanism using isotopic labeling.^43^ They observed that the rate of ^56^Fe dissolution from the electrode matched the rate of ^57^Fe redeposition from the electrolyte, resulting in a constant total Fe content in the electrode. A Fe concentration of 0.01 ppm in the electrolyte was sufficient to maintain this equilibrium, and increasing the Fe^3+^ concentration to 0.1 ppm or higher did not yield further improvements in catalytic activity or stability. Similarly, Heath et al. demonstrated that dissolved Fe can incorporate into Ni, NiO, and NiNiO thin-film electrocatalysts, enhancing OER activity up to the point of Fe supersaturation.^14^ Using energy-dispersive X-ray spectroscopy (EDX) and X-ray photoelectron spectroscopy (XPS), they found that Fe preferentially incorporates at the catalyst surface rather than in the bulk. Bao et al. further supported the dissolution/redeposition mechanism by showing that Fe incorporation contributes to the time-dependent enhancement of OER activity in Ni thin films and that the extent of Fe inclusion, and hence the final catalytic performance, depends on the nature of the starting material.^15^

We hypothesized in Section 3.3 that such a dynamic Fe dissolution/redeposition mechanism is partially responsible for the increase in per-site activity observed for activated NiO + γ-Fe_2_O_3_ catalysts. To further investigate the possibility of dissolved Fe leading to activation, we performed a series of studies on NiO with 20 μM Fe^3+^ added to the electrolyte. This concentration, 20 μM, was selected to mimic the conditions of dissolved Fe participating in the hypothesized dissolution/redeposition mechanism and was calculated as the concentration of Fe that would be present at the near surface of the electrolyte (5% of the total volume) if all the Fe in the catalyst were dissolved. Full details are available in Section S9.

The results of these experiments are shown in Figure 9 as Tafel plots (Figure 9a) at the initial state (green solid line), immediately after Fe^3+^ addition (dashed red line), and after activation via triangle wave cycling (13.5 h, dash-dotted yellow line). At the initial condition, the Tafel plot of NiO has a significant redox feature between 0.1 and 1 mA/cm^2^, which can be attributed to the Ni^2+^/Ni^3+^ transition, as was observed for NiFe_2_O_4_. Without Fe, the NiO sample had an initial J@1.65 of 0.6 ± 0.2 mA/cm^2^ (Figure 9b) that, after activation, reached up to 2.8 ± 0.8 mA/cm^2^ (+280 ± 30%; square wave cycling, Figure S9). Additional results for NiO (without Fe^3+^) after activation are included in Section S9.

**Figure 9:**
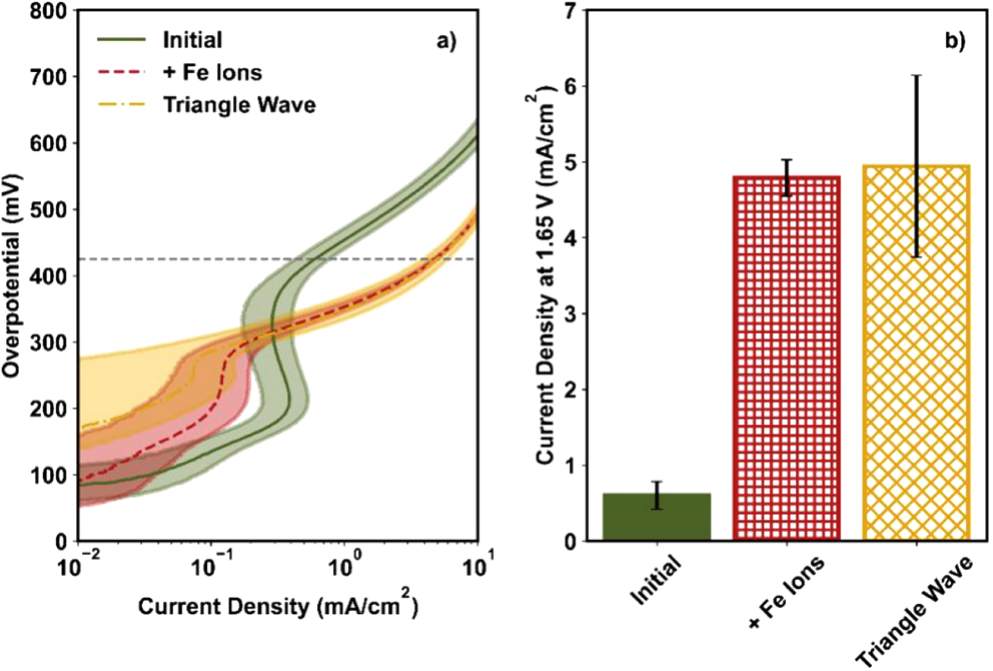
(a) Tafel plot and (b) current density at 1.65 V for NiO with no Fe in the electrolyte, after the addition of 20 μM of Fe^3+^, and after triangle wave cycling (13.5 h). In Figure a, LSVs were collected at a scan rate of 20 mV/s with an RDE rotation rate of 2500 rpm. The dashed gray line is at 1.65 V. The line and the shaded region represent the average and standard deviation, respectively, of three independent experiments. In Figure b, the bar and error bars represent the average and standard deviation, respectively, of three independent experiments.

Upon the addition of Fe^3+^ ions to the electrolyte, the activity improved dramatically. This can be seen in the Tafel plots shown in Figure 9a, where the overpotentials dropped by more than 100 mV, and the J@1.65 increased by nearly 700% to 4.8 ± 0.2 mA/cm^2^, higher than was observed for any other catalyst before or after activation. This result supports the hypothesis that dissolved Fe can incorporate into Ni­(OH)_2_/NiOOH frameworks formed on the surface of NiO and increase the level of the OER activities. Interestingly, after triangle wave cycling, there was no further change in the activity of this material (J@1.65, 4.9 ± 1.1 mA/cm^2^). This result suggests that any changes to the active surface occurred immediately upon the addition of Fe ions and were not further improved by additional potential cycling, in contrast to the results for NiFe_2_O_4_ and NiO + γ-Fe_2_O_3_.

The dramatic enhancement of NiO activity immediately after the inclusion of Fe ions, as well as the lack of additional enhancement after activation, suggests that the limiting factor in the activation of NiFe oxides may be the rate of dissolution of Fe rather than the rate of redeposition. These results suggest that incorporating dissolved Fe into electrolytes by means of activation may be a promising strategy. Such a strategy, however, inevitably raises durability concerns at the device level, as past studies have shown degradation in the presence of dissolved Fe for AEMWEs.^48,49^ Additional exploration on the use of dissolved Fe in catalyst conditioning, but perhaps not in ultimate device performance, is therefore of interest for future work.

## 4 Conclusions

The activation of commercially available and relevant NiFe oxides was probed via three different types of activation procedures: chronoamperometry at 1.8 V, triangle wave cycling from 1.4 to 2.0 V, and square wave cycling from 1.4 to 2.0 V. The results demonstrate that NiFe oxides activate over time on stream regardless of the starting condition (i.e., atomically mixed as in NiFe_2_O_4_, physically mixed as in NiO + γ-Fe_2_O_3_, or with Fe from the electrolyte as in NiO+Fe^3+^(aq)). For NiFe_2_O_4_, activation was directly attributed to the formation of new NiFeOOH active sites (i.e., an increase in the active site quantity). For NiO + γ-Fe_2_O_3_, activation was instead attributed to both an increase in active site quantity (surface roughening, formation of additional Ni­(OH)_2_/NiOOH from NiO) and an increase in active site quality (transformation of Ni­(OH)_2_/NiOOH to NiFe­(OH)_2_/Ni­(Fe)­OOH). Specifically, Ni­(Fe)­OOH formation for this species was hypothesized to occur via a dynamic Fe dissolution/redeposition mechanism, wherein dissolved Fe was incorporated into the formed Ni­(OH)_2_/NiOOH. Furthermore, this work showed that not only does the starting phase of the parent material matter but the method of activation affects both the quantity of active sites formed and the rate of active site formation. These important active phases were found to form quasireversibly on NiFe oxide surfaces, necessitating in situ characterization to understand the behavior of these catalysts during operation in electrochemical devices.

Understanding catalyst changes is critical to advancing catalyst development design principles and ultimately improving kinetics and cell efficiency at the device level (such as for AEMWEs), as well as increasing our understanding of relevant material changes and degradation processes. Such insights and advances are relevant to multiple electrochemical technologies (e.g., AEMWE, PEMWE, liquid alkaline water electrolyzers, AEM and PEM fuel cells, CO_2_ electrolyzers, and batteries) that leverage multicomponent catalysts and have a need to better understand how those elements interact and lead to improved activity.

## Supplementary Material



## Notes

Methods for reference electrode calibration; schematics for activation procedures; ICP-MS and XRD results; results for double layer capacitance estimations; methods for the determination of TOF_redox_; raw experimental data for the extended activation procedures for NiFe_2_O_4_ and for NiO with and without γ-Fe_2_O_3_ or Fe_3_^+^ (CVs, Tafel slopes, and measured exchange current densities before and after activation procedures) (PDF)
