# Supplementary material for: Electrochemical Activation of Ni–Fe Oxides for the Oxygen Evolution Reaction in Alkaline Media
Source: ACS Catal. 2025 Jun 18;15(13):11475–86. doi: 10.1021/acscatal.5c02405 (PMC12235582; doi:10.1021/acscatal.5c02405)
Supplement: Supplementary file 1 [file cs5c02405_si_001.pdf]

Supplementary Information for: Electrochemical activation of NiFe oxides for the oxygen evolution reaction in alkaline media

Emily K. Volk[1], Melissa E. Kreider[2], Daniella M. Gibson Colón[2,3], Magdalena Müller[4], Svein Sunde[4], Shaun M. Alia[2*], Stephanie Kwon[1,5*]

[1]Advanced Energy Systems Graduate Program, Colorado School of Mines, Golden Colorado 80401, United States

[2]Chemistry and Nanoscience Center, National Renewable Energy Laboratory, Golden, Colorado 80401, United States

[3]Department of Chemistry, University of Puerto Rico - Rio Piedras, San Juan, PR, 00925-2537, United States

[4]Department of Materials Science and Engineering, Norwegian University of Science and Technology (NTNU), N-7491, Trondheim, Norway

[5]Department of Chemical and Biological Engineering, Colorado School of Mines, Golden, Colorado 80401, United States

* *Corresponding authors:* shaun.alia@nrel.gov, kwon@mines.edu

S.1 Reference Electrode Calibration

The Hg/HgO reference electrode was calibrated using the HER/HOR reaction on a polycrystalline Pt RDE (0.1963 cm^2, Pine Research Materials). First, to saturate the electrolyte with H_2, H_2 gas was bubbled into the electrolyte for 15 minutes with the Pt disc rotating. Next, the Pt disc was conditioned with 10 cycles of cyclic voltammetry from -0.2 to 1.2 V vs. RHE. Finally, a linear sweep voltammogram was collected from -0.2 to 1.2 V vs. RHE. The correction factor was determined as the point where the HER/HOR curve crossed J = 0.

S.2 Testing Profiles

The three activation test profiles used in this work are included schematically below in **Figure S1**.

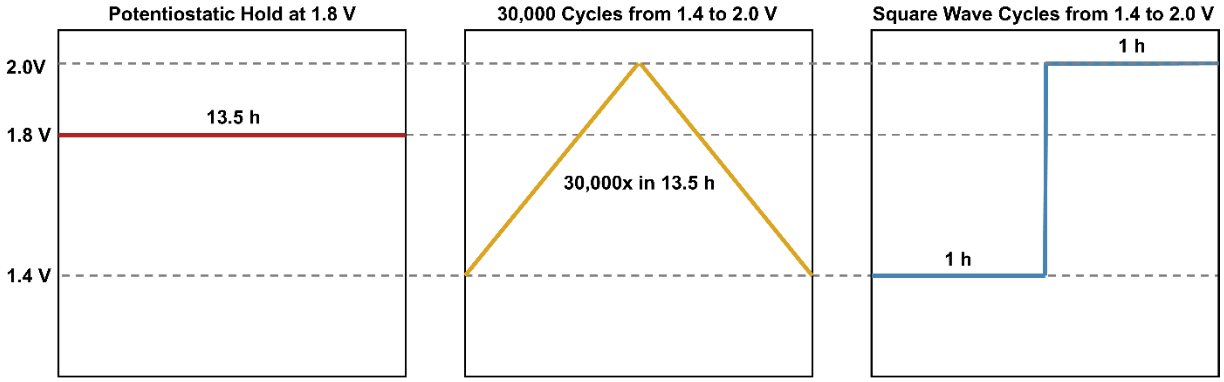

Figure S1. Schematics of the three testing profiles employed in this work.

S.3 ICP-MS and XRD Results

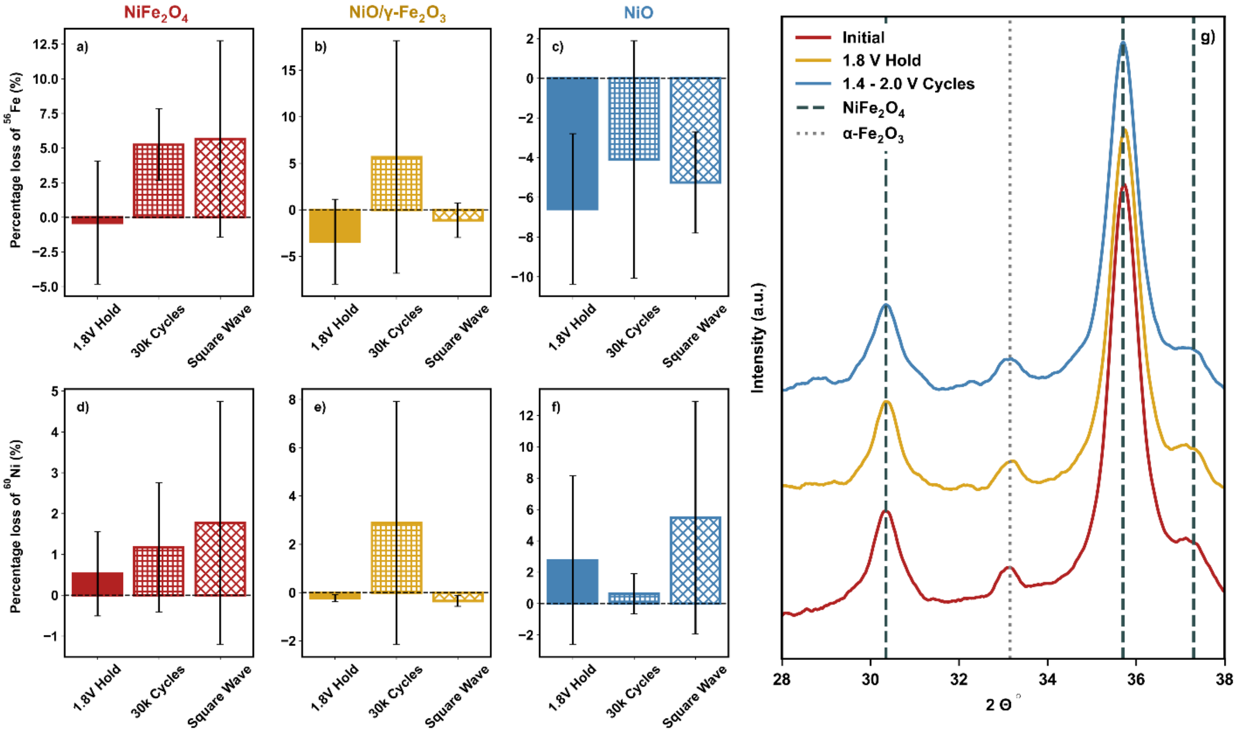

Figure S2. (a-c) Percentage loss of Fe measured from ICP-MS using the ^{56}Fe signal from (a) $NiFe_2O_4$, (b) NiO/γ-Fe_2O_3, and (c) NiO after three activation procedures. (d-f) Percentage loss of Ni measured from ICP-MS using the ^{60}Ni signal from (d) $NiFe_2O_4$, (e) NiO/γ-Fe_2O_3, and (f) NiO after three activation procedures. (g) X-ray diffractograms of $NiFe_2O_4$ before and after two activation procedures.

S.4 Raman experimental profiles

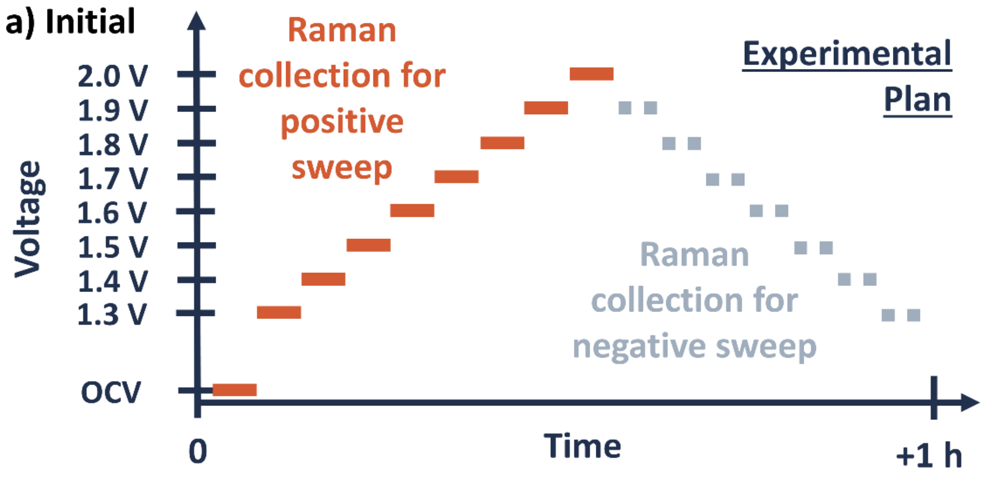

Figure S3. (a) Experimental profile for Raman spectra collection for the "Initial" condition. Voltage was collected at OCV and then at increasing potentials up to 2.0 V for approximately 5 minutes per potential. Spectra were then collected at decreasing potentials down to 1.3 V for approximately 5 minutes per potential.

S.5 Double layer capacitance

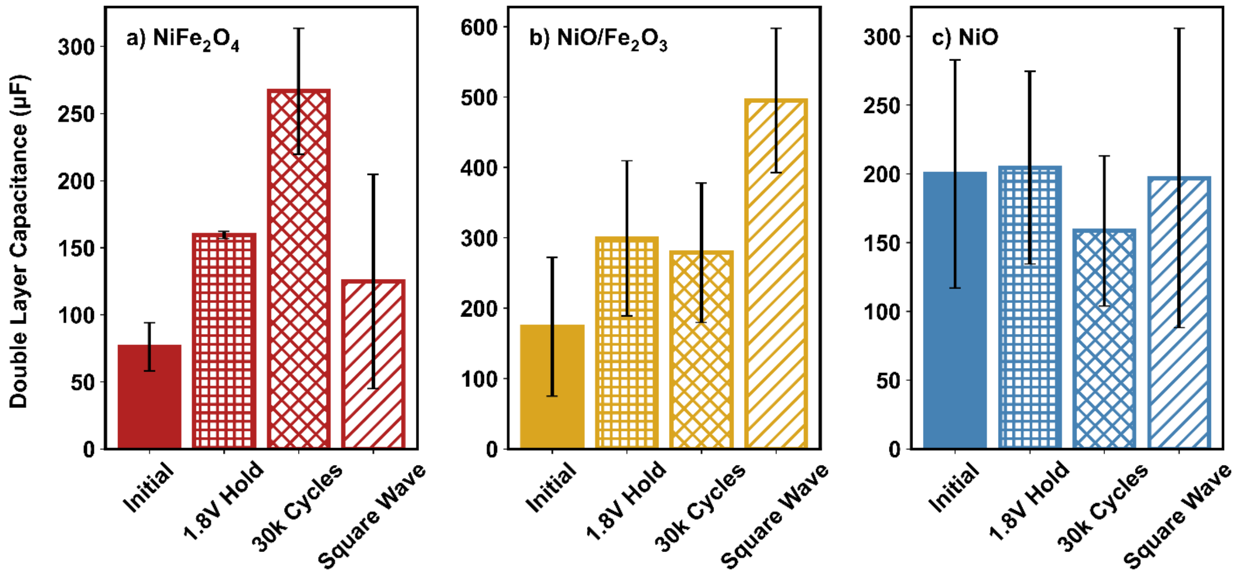

Figure S4. Values for the double layer capacitance before and after different activation procedures for (a) $NiFe_2O_4$, (b) $NiO/\gamma\text{-}Fe_2O_3$, and (c) NiO.

S.6 Determination of TOF_{redox}

The redox TOF can be determined from the following equation:

$$TOF_{redox} = \frac{Molecules\ of\ O_2\ Produced/s}{Molecules\ of\ redox\ active\ Ni}$$

Here, we assume that the Faradaic efficiency is 100% towards OER. The number of molecules of O_2 produced can then be calculated from the current as follows:

$$Moles\ of\ O_2\ produced\ per\ s = \frac{iN_A}{nF}$$

Where i is the current in A, N_A is Avogadro's number, n is the number of electrons transferred in OER, and F is Faraday's constant. Written out:

$$Molecules\ of\ O_2\ Produced\ per\ s$$
$$= \left(i\frac{[C]}{[s]}\right)\left(\frac{mol\ e^-}{96485\ C}\right)\left(\frac{1\ mol\ O_2}{4\ mol\ e^-}\right)\left(\frac{6.022x10^{23}\ molecules}{mol\ O_2}\right)$$

The number of molecules of Ni participating in the Ni^{2+} to Ni^{3+} transition can be calculated from the integration of the redox peak as follows:

$$Molecules\ of\ redox\ active\ Ni = \frac{(A_{red})(N_A)(N_{redox})}{\nu F}$$

Where A_{red} is the area of the reduction peak associated with the Ni^{2+} to Ni^{3+} transition, ν is the scan rate, N_A is Avogadro's number, N_{redox} is the number of moles of electrons transferred per one mole of Ni redox transition, and F is Faraday's constant. Written out:

$$Molecules\ of\ redox\ active\ Ni$$
$$= \left(redox\ peak\ area\frac{VC}{s}\right)\left(\frac{s}{100\ mV}\right)\left(\frac{1000\ mV}{V}\right)\left(\frac{mol\ e^-}{96485\ C}\right)\left(\frac{6.022x10^{23}\ molecules}{mol\ O_2}\right)$$

Here, N_{redox} is assumed to be equal to 1.55 mol e^- / mol redox transition as has been reported previously.[1] Note that this assumes that only the surface Ni is redox-active, which we confirmed by checking the linear dependance between the maximum current of the reduction peak vs. the scan rate for each scan rate. This was found to be linear, validating our assumption.

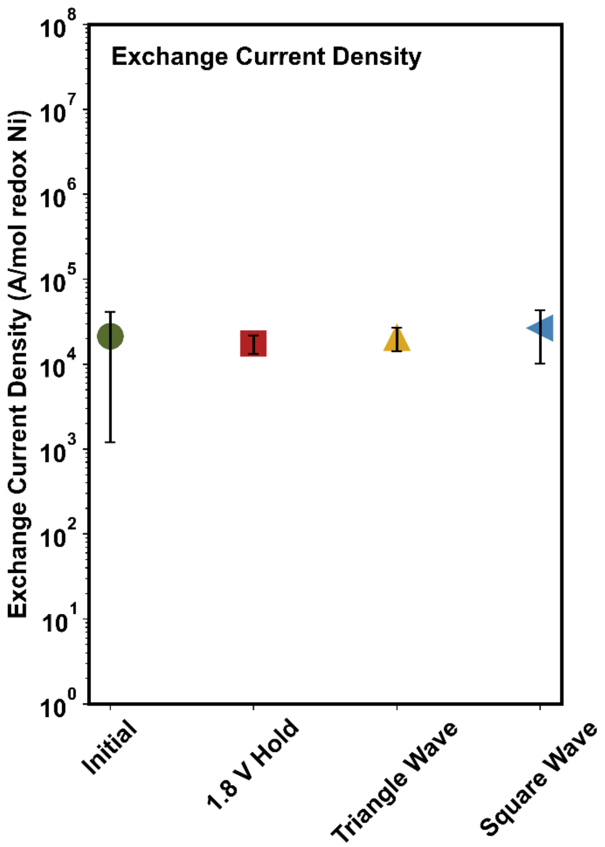

Figure S5. Exchange current densities normalized by the quantity of redox-active Ni (A/mol redox-active Ni) for $NiFe_2O_4$ at the initial state and after three activation procedures.

S.7 Extended activation procedures

The raw Tafel and cyclic voltammetry data for the extended activation tests for $NiFe_2O_4$ are included in **Figure S6**.

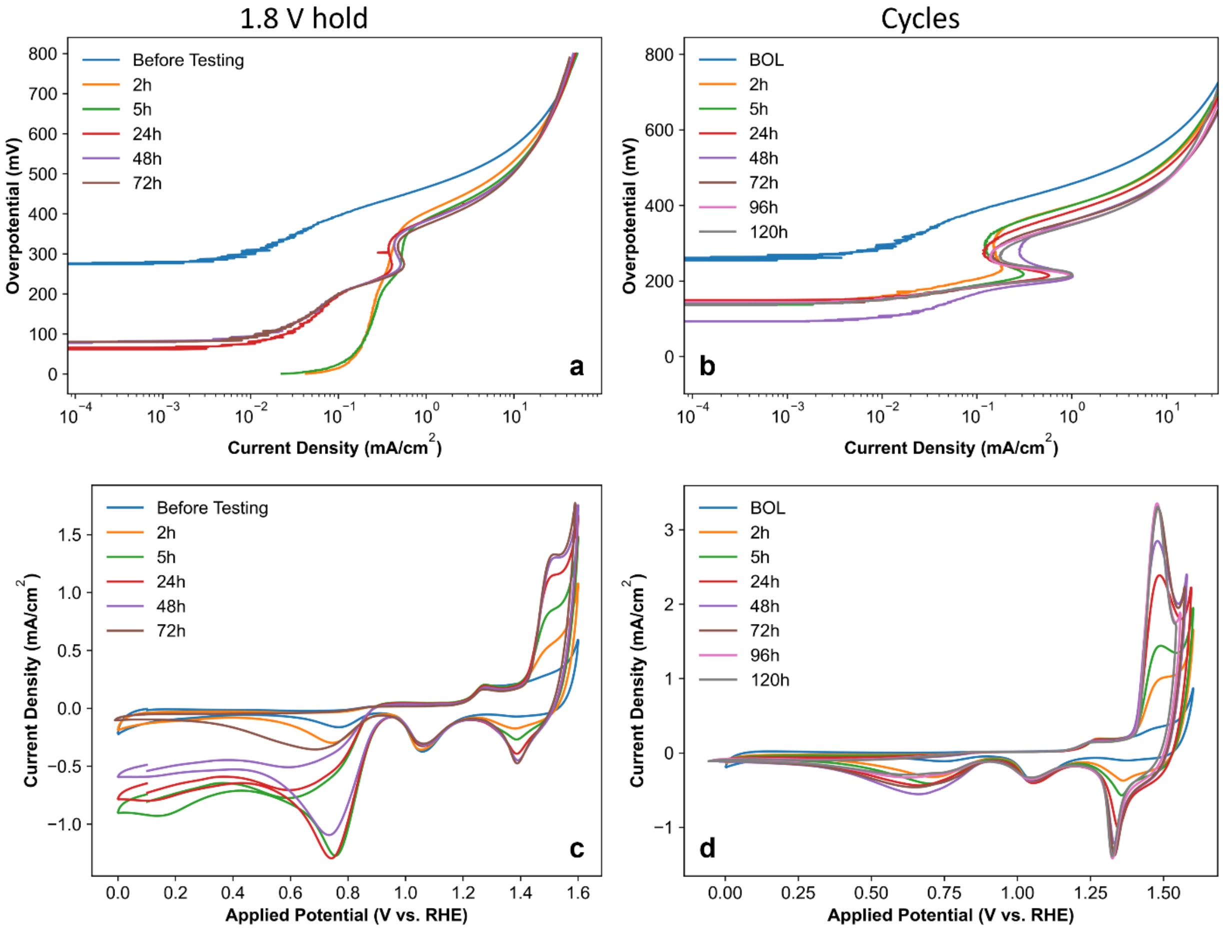

Figure S6. Results for extended activation of $NiFe_2O_4$ during (a, c) 1.8 V hold and (b,d) triangle wave cycling. (a, b) show changes to the Tafel behavior over time, and (c, d) show changes to cyclic voltammograms.

S.8 Additional results for NiO/γ-Fe_2O_3

Like $NiFe_2O_4$, changes to redox features were observed in the cyclic voltammetry results for NiO/γ-Fe_2O_3 after each activation procedure, shown in **Figure S7**. As for $NiFe_2O_4$, features near 0.2 V were present and assigned to the Fe II/III transition. These features, however, were much larger for the NiO/γ-Fe_2O_3 catalyst compared to $NiFe_2O_4$, suggesting that more Fe sites are exposed for the physical mixture compared to the spinel. The reductive peak at 0.8 V that was present for $NiFe_2O_4$ was not distinguishable for NiO/γ-Fe_2O_3, consistent with the lower current densities for this material (and therefore less evolved O_2 to reduce in the cathodic sweep of the voltammogram). Peaks between 1.0 and 1.2 V from the Au substrate were evident. Lastly, there was an increase in the peak height after activation for the redox pair associated with the Ni II/III transition, like the $NiFe_2O_4$ results. This increase, however, was smaller for NiO/γ-Fe_2O_3 than for $NiFe_2O_4$ after any activation procedure, suggesting less exposure of redox-active Ni sites.

We predict that some of the dissolved Fe redeposited in the FeOOH phase (similar to what was described by Kuai et al.[2]), explaining the increase in Fe redox features in cyclic voltammetry (**Fig. S7**).

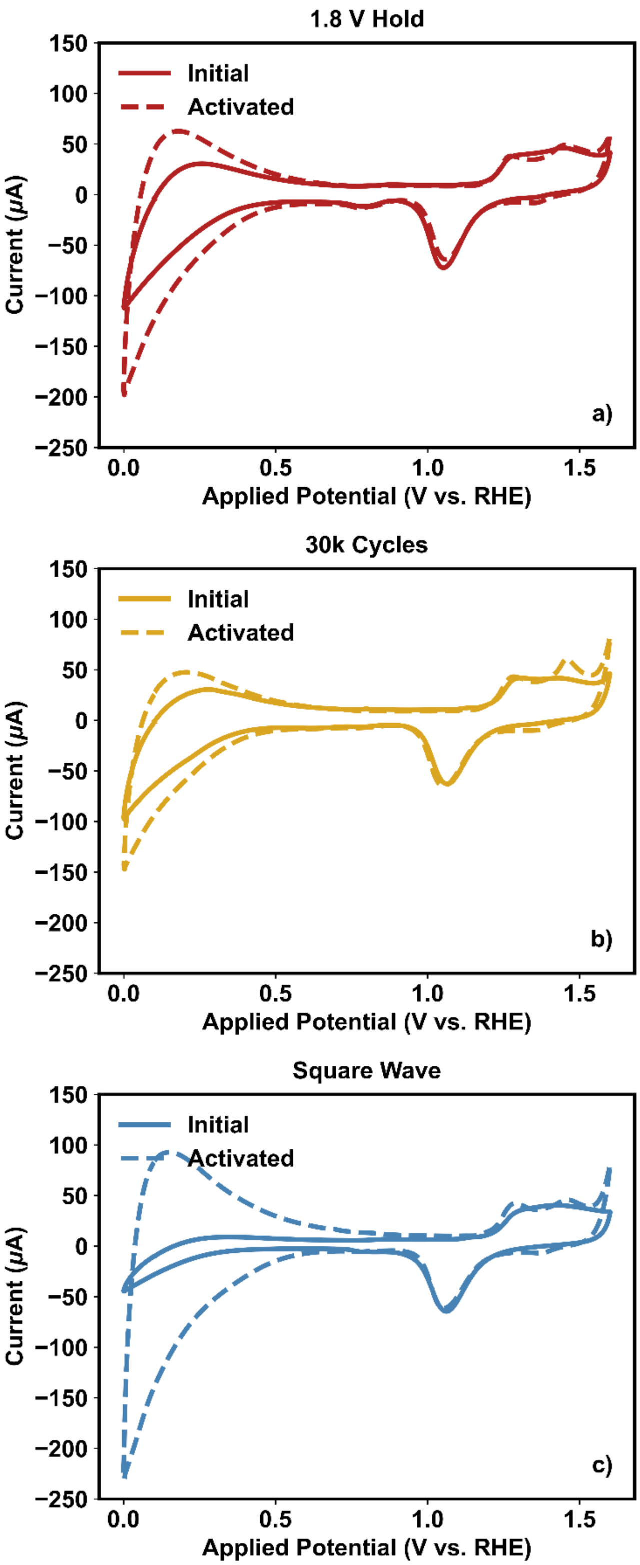

Figure S7. Cyclic voltammograms for NiO/γ-Fe_2O_3 after (a) 1.8 V hold, (b) Triangle wave cycling, and (c) square wave cycling.

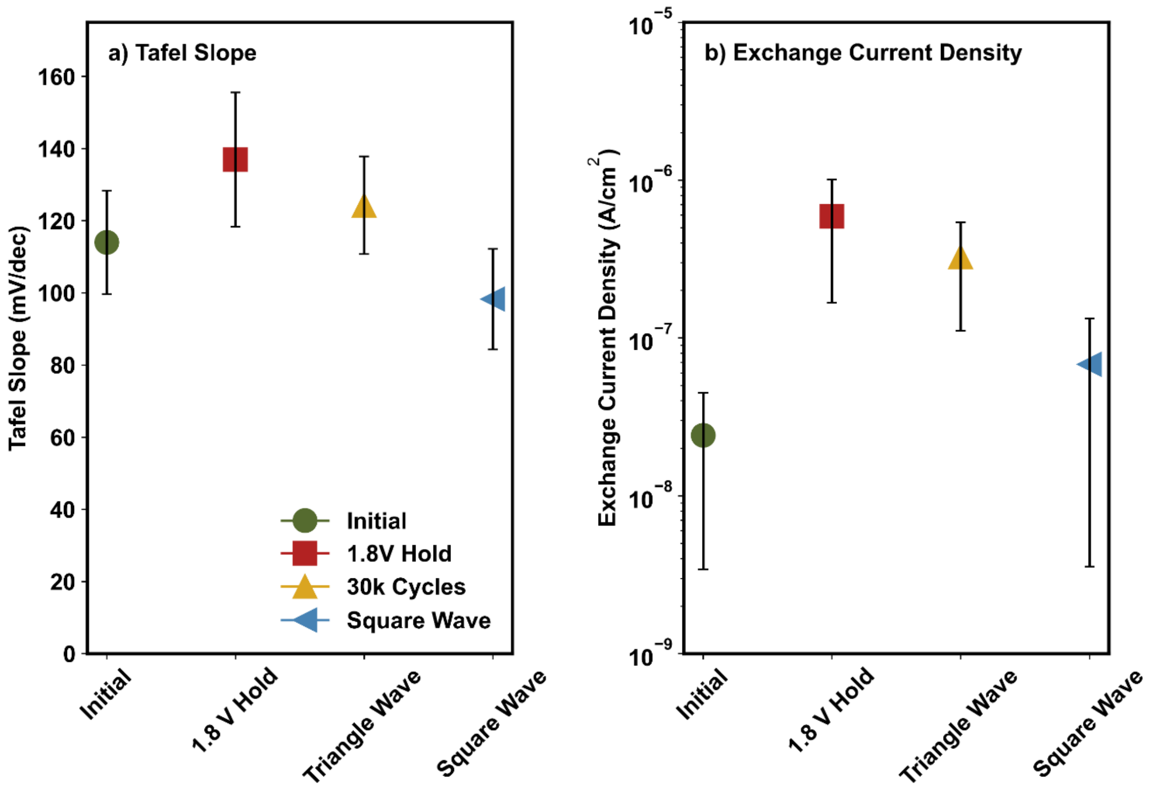

Figure S8. Values for the (a) Tafel slope and (b) exchange current density for NiO+γ-Fe_2O_3 and after three activation procedures.

The Tafel slope for NiO+γ-Fe_2O_3 was largely unchanged within experimental error after activation (**Fig. S8a**). The exchange current density, however, increased after activation, suggesting an increase in the per site activity (**Fig. S8b**).

S.9 Additional electrochemical results for NiO with and without Fe^{3+}

20 µM of Fe3+ ions was selected to mimic the quantity of Fe which would be present in the electrolyte if all of the Fe in the catalyst dissolved. Approximately 20 µg of catalyst was deposited on the catalyst surface. This was converted to moles of Fe using the molecular weight of $NiFe_2O_4$ (234.37 g/mol) and the molar ratio of Ni:Fe in this material (2 mol Fe per mol $NiFe_2O_4$). Next, we assumed that in the hypothesized Fe dissolution / redeposition mechanism, the dissolved Fe would remain at the near surface of the electrode. We therefore quantified the molarity of the electrolyte in 5% of the total volume (i.e., 0.05*130 mL, assuming 5% of the volume to be the near-surface volume) and found this to be approximately 20 µM.

The activity of NiO was found to increase after activation in all cases (**Fig. S9**), concurrent with an increase in the intensity of the Ni^{2+} to Ni^{3+} transition (**Fig. S10**), consistent with the electrochemical aging of NiO to $Ni(OH)_2$/NiOOH.[3]

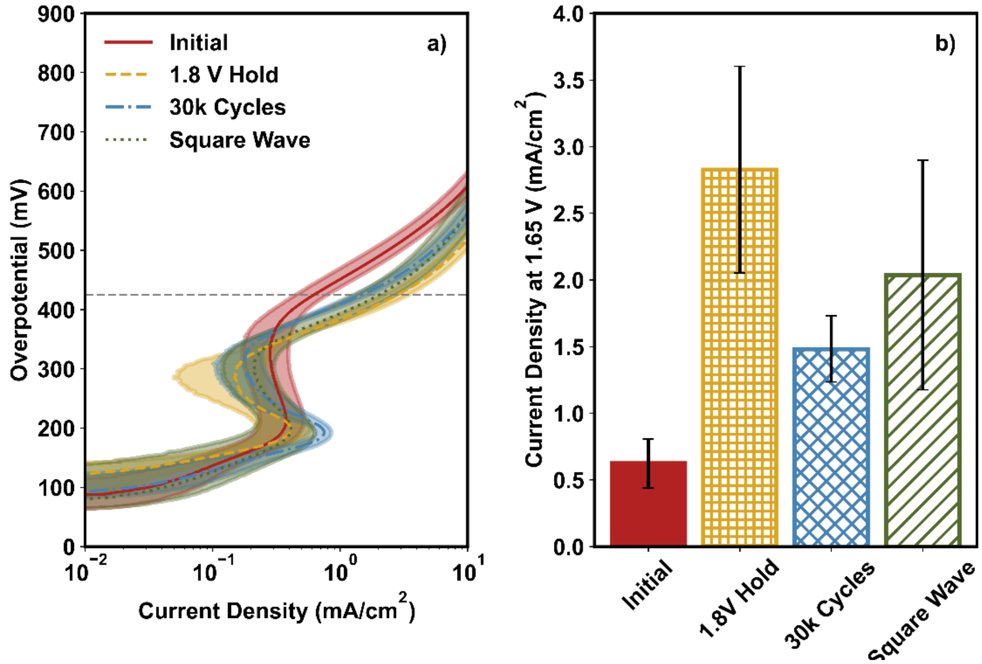

Figure S9. a) Tafel plot and b) current density at 1.65 V before and after three different activation procedures for NiO. In a), LSVs were collected at a scan rate of 20 mV/s with an RDE rotation rate of 2500 RPM. The dashed grey line is at 1.65 V. In a), the line represents the average of three experiments, and the shaded region represents the standard deviation between them. In b), the bar and error bars represent the average and standard deviation, respectively, of three experiments.

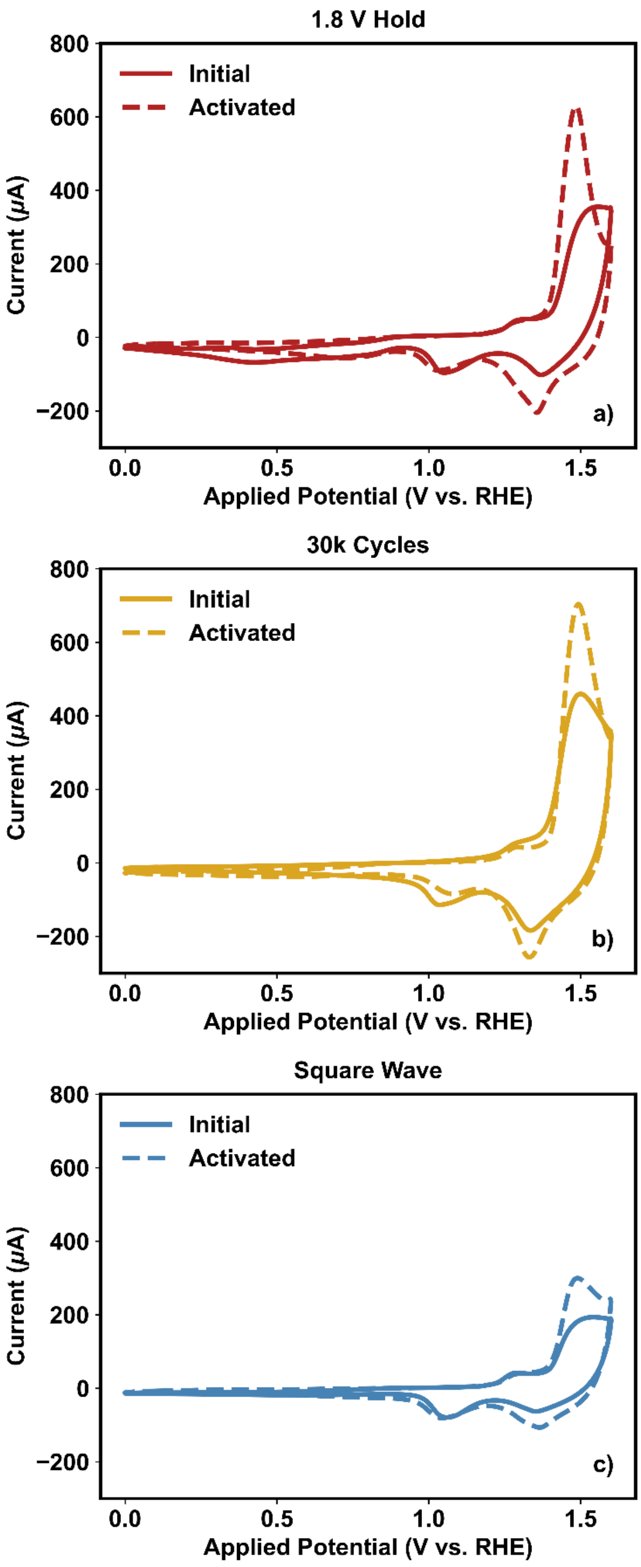

Figure S10. Cyclic voltammograms for NiO after (a) 1.8 V hold, (b) Triangle wave cycling, and (c) square wave cycling.

(1) Mattinen, M.; Schröder, J.; D'Acunto, G.; Ritala, M.; Jaramillo, T. F.; Stevens, M. B.; Bent, S. F. Dynamics of Precatalyst Conversion and Iron Incorporation in Nickel-Based Alkaline Oxygen Evolution Reaction Catalysts. *Cell Reports Physical Science* **2024**, *0* (0). https://doi.org/10.1016/j.xcrp.2024.102284.

(2) Kuai, C.; Xu, Z.; Xi, C.; Hu, A.; Yang, Z.; Zhang, Y.; Sun, C.-J.; Li, L.; Sokaras, D.; Dong, C.; Qiao, S.-Z.; Du, X.-W.; Lin, F. Phase Segregation Reversibility in Mixed-Metal Hydroxide Water Oxidation Catalysts. *Nat Catal* **2020**, *3* (9), 743–753. https://doi.org/10.1038/s41929-020-0496-z.

(3) Bode, H.; Dehmelt, K.; Witte, J. Zur Kenntnis Der Nickelhydroxidelektrode—I.Über Das Nickel (II)-Hydroxidhydrat. *Electrochimica Acta* **1966**, *11* (8), 1079–1087. https://doi.org/10.1016/0013-4686(66)80045-2.
